# Dual oncogenic role of RNF220 in AML: linking metabolic rewiring to cell proliferation and immune evasion

**DOI:** 10.3389/fonc.2025.1670895

**Published:** 2025-10-30

**Authors:** Bixia Li, Shi Jiang, Yao Xu, Xiao Yan, Qitian Mu, Guifang Ouyang

**Affiliations:** ^1^ Department of Hematology, The First Affiliated Hospital of Ningbo University, Ningbo, China; ^2^ Department of Breast Surgery, The First Affiliated Hospital of Ningbo University, Ningbo, China; ^3^ Department of Medical Equipment, Ningbo Medical Center Lihuili Hospital, Ningbo, China

**Keywords:** acute myeloid leukemia, RNF220, tumor metabolism, tumor microenvironment, immune evasion

## Abstract

**Background:**

Acute myeloid leukemia (AML) remains a clinical challenge with suboptimal long-term survival. While circular RNAs derived from the RNF220 host gene have been implicated in AML pathogenesis, the functional role and regulatory mechanisms of RNF220 itself in AML are poorly understood.

**Methods:**

We integrated bioinformatics analyses of public databases (TCGA-LAML, TARGET-LAML) and local cohort with *in vitro* functional assays. RNF220 was knocked down and overexpressed in AML cell lines using lentivirus. Transcriptomic profiling (RNA-seq), metabolic pathway enrichment (GSVA, GSEA), and immune microenvironment deconvolution (xCELL, CIBERSORT, MCP-counter) were performed. Transcription factor binding sites were predicted across five databases (JASPAR, ENCODE, GTRD, etc.). Validation of transcriptional regulation was performed using ChIP-PCR and luciferase reporter assays.

**Results:**

RNF220 overexpression correlated with poor prognosis in AML, drove an immunosuppressive microenvironment characterized by reduced CD8^+^ T cells, and inhibited NK activity and M2 polarization of macrophage. RNF220 promoted tumor proliferation by suppressing apoptosis and preventing G1 arrest. Knockdown of RNF220 dysregulated metabolic pathways, notably suppressing glycolysis and phenylalanine metabolism. Mechanistically, FOXA1 was identified as an upstream negative regulator of RNF220, where high FOXA1 predicted favorable survival and inversely correlated with RNF220-associated metabolic reprogramming.

**Conclusion:**

NF220 acts as an oncogenic ubiquitin ligase in AML by coordinating dual pro-leukemic mechanisms: cell-intrinsic metabolic rewiring (glycolysis/phenylalanine) and immune evasion via microenvironment suppression. Targeting the FOXA1–RNF220 axis may offer novel therapeutic strategies for high-risk AML.

## Background

Acute myeloid leukemia (AML) comprises a relatively well-defined group of hematopoietic neoplasms involving precursor cells committed to the myeloid lineage. AML is the most common type of acute leukemia in adults, accounting for approximately 80% of acute leukemia cases (1, 2). The incidence of AML in adults is approximately 3–5 cases per 100,000 individuals (3–5).

In recent years, with continuous advancements in medical technology, including the development of novel targeted therapies, improvements in hematopoietic stem cell transplantation techniques, and the clinical application of immunotherapy, the clinical efficacy of AML treatment has significantly improved. Nevertheless, the long-term survival rate for adult non-M3 (non-acute promyelocytic leukemia) AML patients remains below 40% (6).

Several factors constrain the clinical efficacy of AML treatment. Firstly, for the majority of AML patients, the driver genes and key signaling pathways crucial for normal cellular development and differentiation remain incompletely understood. The inability to identify effective therapeutic targets hinders the development of targeted drugs. Secondly, while cytogenetic karyotype analysis is one of the most effective prognostic markers in AML, patients with cytogenetically normal AML constitute 40%–50% of cases. Although molecular mutations, such as FLT3-ITD, NPM1, CEBPA, and CKIT, have been incorporated into the AML risk stratification system alongside karyotype (7, 8), the prognosis of intermediate-risk patients still exhibits substantial heterogeneity, which complicates the selection of optimal clinical treatment strategies (9).

Therefore, the primary tasks for improving the cure rate of adult AML remain elucidating the molecular pathogenesis of the disease, identifying effective therapeutic targets, and refining the prognostic risk assessment system.

Ubiquitination is the most common intracellular pathway regulating protein degradation, and alterations in ubiquitination regulation play a critical role in modulating tumor proliferative capacity (10–12). During ubiquitination, E3 ligases serve as essential factors that recognize diverse substrates and determine the specificity of ubiquitination (13). RING finger proteins (RFPs), a subclass of zinc finger proteins, represent an important class of E3 ligases characterized by a C3HC4-type amino acid motif capable of binding zinc ions (14). Based on subunit composition, RING finger family proteins are classified as either monomeric or multi-subunit complexes. Multi-subunit complexes contain two or more protein subunit domains, with at least one harboring a RING finger domain (15, 16). The RING finger family is extensive, recognizes a wide array of substrates, and plays significant roles in tumor initiation and progression.

RNF220 is a member of the RFP family. Depending on the isoform, its amino acid length ranges from 566 to 592 residues, with the characteristic RING finger domain located between residues 514 and 553. Currently, reports on RNF220 are relatively limited. One study demonstrated that RNF220 can specifically bind to the SIN3B protein and mediate its ubiquitin-dependent degradation (17). However, this report only validated the role of RNF220 at the molecular interaction level and did not extend to cellular or clinical levels to elucidate its function.

Beyond this, literature concerning RNF220 primarily focuses on its role as an E3 ligase in embryonic neural development. Studies indicate that RNF220, by specifically degrading key transcription factors including DBX1/2 and NKX2.2, induces the generation of visceral motor neurons and somatic motor neurons in the ventral spinal cord. Its ability as an E3 ligase to ubiquitinate and degrade target proteins is modulated by its co-factor ZC4H2 (18). The ZC4H2–RNF220 complex can ubiquitinate and degrade Gli proteins, ensuring proper neural development in the ventral spinal cord (19). In recent years, emerging evidence has begun to implicate RNF220 in tumorigenesis (20, 21). In bladder cancer, RNF220’s m6A modification induces cisplatin resistance and immune evasion through K48-linked ubiquitination of PDE10A (22). In medulloblastoma, RNF220 facilitates tumor proliferation and progression by activating the Sonic Hedgehog signaling pathway (23). In leukemia, it was reported that RNF220 promotes the proliferation of leukemic cells and reduces the degradation of the CyclinD1 protein through USP22, which indicated that RNF220 might play an important role in the progression of AML (24).

Circular RNAs (circRNAs) have been reported in recent years to participate in multiple processes of tumorigenesis and progression and are now recognized as significant components in oncology research (25). Notably, numerous circRNAs are reported to function through their host genes (26–28). In a previously published study by our research group, circ_0012152—a circRNA derived from the RNF220 host gene—was found to play an important role in AML via the miR-652-3p/SOX4 axis (29). Concurrently, literature also reports that circ_0012152 promotes AML proliferation through miR-330-5p/SOX4 (30). Furthermore, in AML, circ_0012152 can also enhance proliferation via the miR-30/MYSM1/IER2 axis (31), indicating its significant role in the disease. However, the function and underlying mechanisms of its host gene, RNF220, in AML remain largely unexplored. However, the function of its host gene RNF220 in AML has not yet been reported. Therefore, this study aims to provide a detailed characterization of the role of RNF220 in AML. Based on this, we analyzed the role of RNF220 in AML through online datasets and samples from local patients and confirmed via *in vitro* experiments that RNF220 promotes the proliferation of AML cells and alters their energy metabolism. Bioinformatics analysis further suggested that RNF220 may influence the immune microenvironment by modulating metabolic products. These findings provide valuable insights for the clinical diagnosis and treatment of AML.

## Method

### AML samples and patients

Bone marrow mononuclear cells were obtained from 94 individuals diagnosed with AML. These samples were stored at The First Affiliated Hospital of Ningbo University. The karyotypes of all patients were determined according to the 2017 European LeukemiaNet classification of AML (32). The inclusion criteria were as follows: bone marrow samples from AML patients stored in the local biobank, collected between 1 January 2010 and 31 December 2015, with sufficient material for quantitative PCR (qPCR) analysis. The exclusion criteria included 1) patients with other primary malignant tumors; 2) those who died during the follow-up period due to causes unrelated to the target disease; 3) patients with severe comorbidities (e.g., significant cardiac, hepatic, or renal dysfunction); 4) pregnant or lactating women (if treatment or disease progression could be affected); and 5) samples with improper fixation, severe autolysis, or degradation. The study was approved by the Ethics Committee of the First Affiliated Hospital of Ningbo University (Ningbo First Hospital, China) and was conducted in compliance with relevant medical ethics regulations. Informed consents were obtained from all subjects or their legal guardians.

### Cell lines and cell maintenance

The AML cell lines MV4-11, MOLM13, and THP-1 were obtained from the Zhejiang Provincial Key Laboratory of Hematopoietic Malignancy. The cells were cultured in Iscove’s modified Dulbecco’s medium or Dulbecco’s modified Eagle’s medium (Gibco, USA) supplemented with 10% fetal bovine serum (Gibco) at 37°C in an incubator with 5% carbon dioxide. HEK293T cell line was brought from PROCELL (Wuhan, China). HEK293T cell was cultured in Dulbecco’s modified Eagle’s media (DMEM, Invitrogen, Grand Island, NY, USA), with 10% FBS, 100 μg/mL of streptomycin, and 100 U/mL of penicillin and maintained at 37°C in a 5% CO_2_ condition. All cell lines were routinely tested for mycoplasma contamination.

Lentiviruses for short hairpin RNAs (shRNF220-1/2/3), RNF220 overexpression, and corresponding negative control were designed by GenePharma (China). Lentiviral transduction of cell lines was performed according to the manufacturer’s protocol. siRNA transfection was performed using the Lipo3000 transfection reagent system (Yeasen, China). The sequences of the shRNAs and siRNAs are provided in **Supplementary Table S1**.

### qRT-PCR

Total RNAs from AML samples were extracted using TRIzol reagent (Ambion, USA). Complementary DNA (cDNA) was synthesized using Hifair^®^ II 1st Strand cDNA Synthesis SuperMix for the qPCR Kit (Yeasen, China). qPCR was performed using the Hieff UNICON^®^ qPCR SYBR Green Master Mix (Yeasen, China). Three independent replicates were carried out with every experiment. The ΔΔCt method was used to calculate the relative quantification of mRNA. Glyceraldehyde 3-phosphate dehydrogenase (GAPDH) was used as internal control. Sequences of primers used in this study are depicted in **Supplementary Table S1**.

### Western blot

RIPA lysis buffer was used to extract the total protein. Sodium dodecyl sulfate-polyacrylamide gel electrophoresis was used to separate the extracted protein, and the PVDF membrane (Millipore, USA) was used to transfer protein. After that, the transferred membrane was incubated with an appropriate antibody overnight at 4°C. The next day, enhanced chemiluminescence reagents (FDbio Science, China) were used to detect the antigen–antibody complex on the membrane.

### *In vitro* cell proliferation and lactic acid assay

For the cell counting kit-8 assay, a total of 3 * 10^3^ cells with 100 μL medium were seeded into each well in 96-well plates. Then, the seeded plates were incubated in a humidified 5% CO_2_ atmosphere at 37°C for 1, 2, 3, and 4 days separately. Ten microliters of the CCK-8 solution was added to each well, followed by another 2-h incubation period. The absorbance was then measured using a 96-well plate reader at 450 nm. Lactate levels were measured using the Lactate Assay Kit (No. E-BC-K044-M, Elabscience, Wuhan, China) according to the manufacturer’s instructions. All experiments were performed with three biological replicates. Three replicates were conducted in each experiment.

### Apoptosis assay

The Annexin V-PE/7-AAD apoptosis kits (MULTI SCIENCES, Hangzhou, China) were used to determine cell apoptosis. Infected cells were washed with prechilled phosphate-buffered saline (PBS) and then resuspended in 500 µL of 1X binding buffer. The resuspended cells were incubated at room temperature for 15 min in the dark after being added with 10 µL of PI and 5 µL of annexin V-FITC. A BD LSRFortessa cell analyzer (BD Biosciences, USA) was used to analyze the samples. The FlowJo software was used to analyze the raw data.

### Cell cycle analysis

Infected cells were resuspended in PBS, and after centrifugation, DNA-staining solution (MULTI SCIENCES, Hangzhou, China) was added to the tube and incubated for 30 min in the dark at room temperature. The BD LSRFortessa cell analyzer (BD Biosciences, USA) was used to analyze the samples. The FlowJo software was used to analyze the raw data.

### Transcriptome RNA sequencing assay

RNA-seq was performed by Novogene, Beijing, China, according to the following steps: 1) The cells were collected after lentivirus infection; 2) total RNA was extracted; 3) mRNA was enriched; 4) double-stranded cDNA was synthesized; and 5) the data were sequenced and analyzed.

### Chromatin immunoprecipitation and CHIP-PCR assay

Using a CHIP Assay Kit (P2080, Beyotime, China), chromatin immunoprecipitation (CHIP) assays were performed according to the manufacturer’s instructions. Cells were collected and fixed with 1% formaldehyde at 37°C for 10 min, followed sequentially with SDS lysis, DNA shearing, DNA and protein immunoprecipitation, cross-linked DNA reversal, and DNA purification. Real-time PCR assays and qPCR were used to detect the immunoprecipitated DNA fragments. The negative control is the normal rabbit IgG.

### Luciferase assays

The RNF220 mutant promoter and non-mutant promoter were both purchased from General Biol (Anhui, China). After HEK293T cells were seeded into 6-well tissue plates, Lipo3000 (Yeasen, China) was used to transfect siRNA and plasmids 8 h later. Dual-Luciferase Assay (Promega) was used to measure luciferase activity. All experiments were performed in triplicate.

### Bioinformatics and statistical analysis

#### Bioinformatics analysis

Bulk RNA-seq data analysis was performed using the Sangerbox platform (33) (v3.0), leveraging its integrated modules for:

- Correlation analysis
- GSEA/GSVA enrichment analysis
- Kaplan–Meier survival analysis
- Immune cell infiltration analysis
- Pathway enrichment analysis (KEGG, GO, and HALLMARK gene sets)

Standardized pan-cancer datasets—TCGA, TARGET, and GTEx (PANCAN, *N* = 19,131)—were downloaded from the UCSC Xena browser (https://xenabrowser.net/), and further analysis was conducted on selected subsets including TCGA-LAML (*N* = 214), TARGET-LAML (*N* = 142), TARGET-ALL (*N* = 86), and TARGET-ALL-R (*N* = 99).

For immune infiltration estimation, gene expression profiles were extracted for each tumor sample, mapped to GeneSymbol identifiers, and subsequently analyzed using the R package IOBR (version 0.99.9, available at https://www.ncbi.nlm.nih.gov/pmc/articles/PMC8283787/). The following deconvolution methods were applied:

deconvo_mcpcounter (34)
deconvo_xCell (35)
deconvo_CIBERSORT (36)

Differential gene expression analysis was conducted with the limma R package (version 3.40.6). Data were log2-transformed, and a multiple linear regression model was fitted using the lmFit function, followed by empirical Bayes moderation of standard errors with the eBayes function to compute moderated t-statistics, moderated F-statistics, and log-odds of differential expression.

For gene set enrichment analysis (GSEA), a software (version 3.0) was obtained from the GSEA website (http://software.broadinstitute.org/gsea/index.jsp). Samples were divided into two groups based on RNF220/FOXA1 expression levels. Predefined gene sets were used to evaluate enrichment differences, with the following parameters: minimum gene set size = 5, maximum gene set size = 5,000, and 1,000 permutations. A *p*-value <0.01 was considered statistically significant.

Functional enrichment analysis of gene sets was performed using the R package org.Hs.eg.db (version 3.1.0) for GO annotations and KEGG gene annotations obtained via the KEGG REST API (https://www.kegg.jp/kegg/rest/keggapi.html). The clusterProfiler R package (version 3.14.3) was used for enrichment analysis, with gene sets mapped against the background reference. Parameters were set as follows: minimum gene set size = 5 and maximum gene set size = 5,000, and a *p*-value <0.01 was considered statistically significant.

Single-cell RNA sequencing analysis was performed on the CancerSEA database (http://biocc.hrbmu.edu.cn/CancerSEA/) and TISCH2 database (http://tisch.comp-genomics.org/).

#### Statistical analysis

The 2^−ΔΔCT^ method of relative quantification was used to analyze the RNF220 expression of samples from MV4–11 cells and patients with AML. Prognostic and regression analyses of the local cohort were performed using SPSS (version 22.0). Statistical significance was determined using SPSS software (version 22.0; IBM Corporation, USA). Differences in the distribution of continuous variables between groups were identified using the Mann–Whitney *U* test or the *t*-test, and differences between categorical variables were analyzed using the chi-square test. Survival analyses were performed using the Kaplan–Meier method, with comparisons made using the log-rank test. Multivariate analyses were conducted using the Cox proportional hazards regression model. All the statistical tests were performed with 95% confidence intervals (CIs). A *p*-value <0.05 was considered to indicate statistical significance.

## Results

### RNF220 suggests a poor prognosis in leukemia

**Figure 1:**
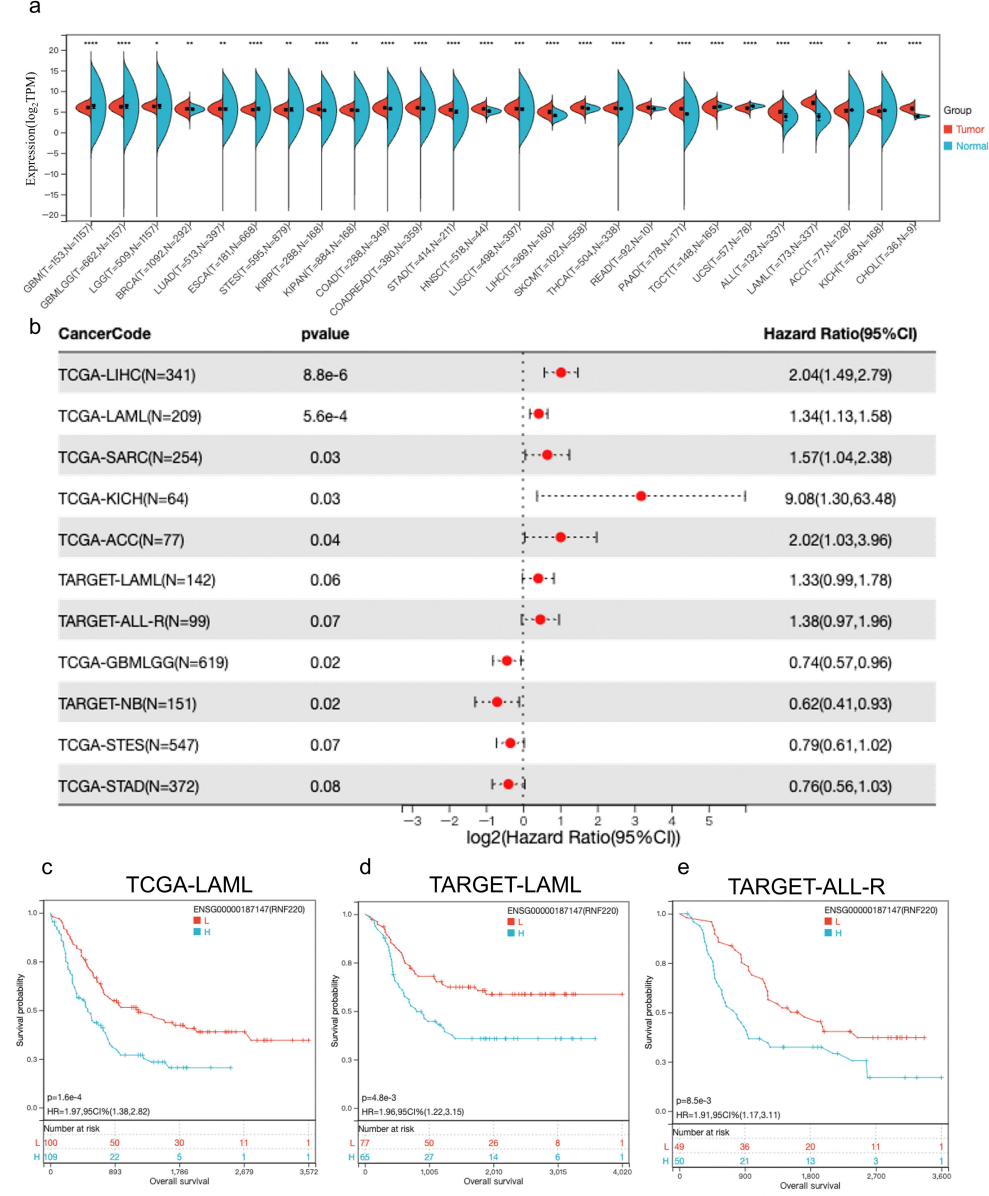
RNF220 suggests poor prognosis in leukemia. **(a)** Expression of RNF220 in tumor and normal tissue from the TCGA database. This panel displays only cancer types with statistically significant differences. **(b)** Forest plot illustrating the impact of RNF220 on overall survival (OS) across various cancers. Only cancer types with *p* <0.1 are displayed. **(c)** Kaplan–Meier curve showing the prognostic impact of RNF220 on OS in the TCGA-LAML dataset. **(d)** Kaplan–Meier curve demonstrating the prognostic impact of RNF220 on OS in the TARGET-LAML dataset. **(e)** Kaplan–Meier curve depicting the prognostic impact of RNF220 on OS in the TARGET-ALL-R dataset. Statistical analyses used non-paired Wilcoxon rank sum and signed rank tests and Kaplan−Meier survival analysis. *p < 0.05, **p < 0.01, ***p < 0.001, and ****p < 0.0001.

Analysis of pan-cancer RNA sequencing data from The Cancer Genome Atlas (TCGA) showed that RNF220 was upregulated in most tumor tissues, with particularly significant upregulation observed in both AML and acute lymphoblastic leukemia (ALL) (**Figure 1a**). Subsequent pan-cancer prognostic analysis indicated that high RNF220 expression was an adverse prognostic factor in most malignancies (**Figures 1b**, **S2a–h**). Further survival analysis confirmed that high RNF220 expression was significantly associated with poor prognosis in both AML and ALL patients (**Figures 1c–e**).

### RNF220 correlates with diverse tumor biological behaviors

**Figure 2:**
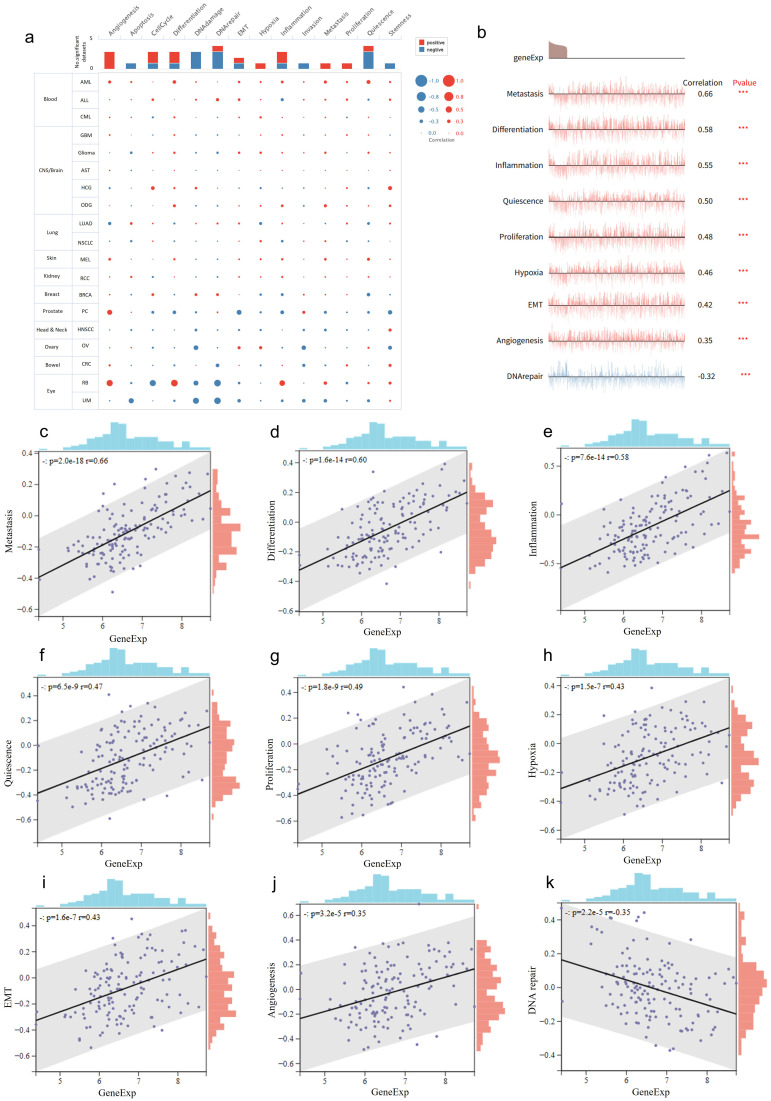
RNF220 correlates with diverse tumor biological behaviors. **(a)** Dots plots depicting the correlations between RNF220 and various oncogenic signaling pathways across multiple single-cell datasets from different cancer types. **(b)** Line map illustrating correlations between RNF220 and oncogenic signaling pathways in diverse single-cell datasets. **(c–k)** Scatter plots demonstrating associations with distinct oncogenic processes. Corresponding signaling pathways are labeled on the *y*-axis. Pearson correlation analysis was performed. ***p < 0.001.

We analyzed the association of RNF220 with various tumor biological functions using single-cell RNA sequencing datasets from the CancerSEA database (http://biocc.hrbmu.edu.cn/CancerSEA/) (AML datasets: Exp0047, Exp0048, Exp0049; ALL dataset: EXP0046; CML dataset: EXP0050). This analysis revealed strong positive correlations between RNF220 expression and key biological processes in hematological malignancies, including tumorigenesis, apoptosis, differentiation, and epithelial–mesenchymal transition (EMT). This positive correlation was especially pronounced in AML (**Figure 2a**). Further correlation analysis demonstrated that RNF220 expression showed the most significant positive correlations with pathways related to metastasis, differentiation, and inflammation, while exhibiting a significant negative correlation with DNA repair pathways (**Figures 2b–k**). Additionally, analysis of three AML and three ALL single-cell datasets from the TISCH2 database (http://tisch.comp-genomics.org/) showed that RNF220 expression was generally higher in malignant cells compared to other cell types, and this trend was more evident in AML (**Supplementary Figures S2a–f**).

### RNF220 is associated with tumor immune evasion

**Figure 3:**
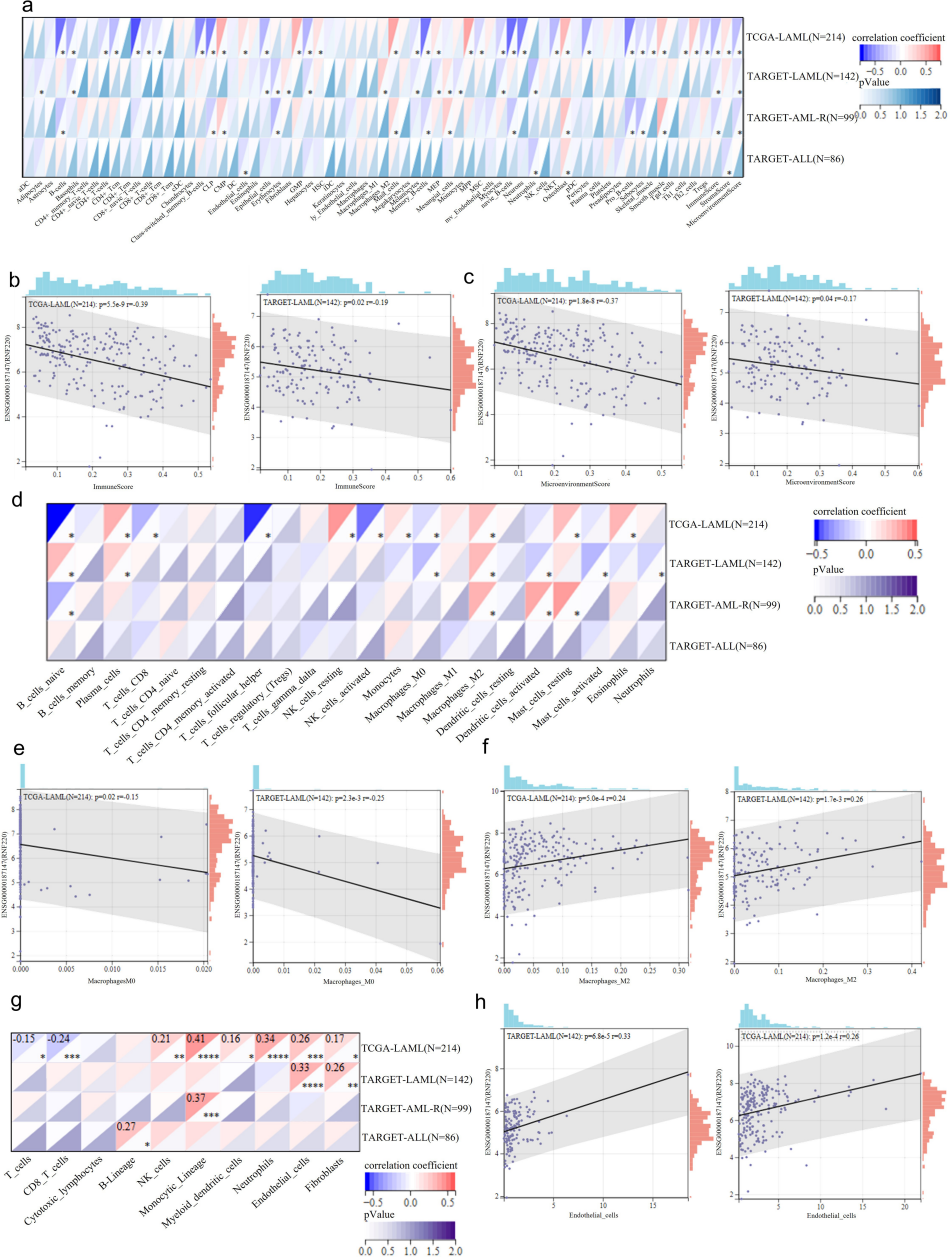
RNF220 is associated with tumor immune evasion. **(a)** Heatmap showing correlations between RNF220 expression and immune cell subtypes analyzed by xCELL algorithm in the TCGA- and TARGET-LAML/ALL datasets. **(b)** Scatter plot demonstrating correlation between RNF220 and immune scores in the TCGA- and TARGET-LAML datasets. **(c)** Scatter plot indicating correlation between RNF220 and tumor microenvironment scores in the TCGA- and TARGET-LAML datasets. **(d)** Heatmap displaying correlations between RNF220 and immune cell subtypes analyzed by CIBERSORT in the TCGA- and TARGET-LAML/ALL datasets. **(e)** Scatter plot showing correlation between RNF220 and M0 macrophage infiltration in the TCGA- and TARGET-LAML datasets. **(f)** Scatter plot illustrating correlation between RNF220 and M2 macrophage infiltration in the TCGA- and TARGET-LAML datasets. **(g)** Heatmap presenting correlations between RNF220 and immune cell subtypes analyzed by MCP-counter in the TCGA- and TARGET-LAML/ALL datasets. **(h)** Scatter plot depicting the correlation between RNF220 and endothelial cell abundance in the TCGA- and TARGET-LAML datasets. Pearson correlation analysis was performed. *p < 0.05, **p < 0.01, ***p < 0.001, and ****p < 0.0001.

To investigate the relationship between RNF220 and the tumor immune microenvironment, we first employed the xCELL algorithm to analyze RNA-seq data from AML and ALL patients in the TCGA and TARGET cohorts, assessing correlations between RNF220 expression and immune cell subtype proportions. We found that the correlation of RNF220 with the immune microenvironment was significantly stronger in AML than in ALL (**Figure 3a**). Although the specific immune cell types correlating with RNF220 differed between the TCGA and TARGET datasets, RNF220 expression consistently showed significant negative correlations with both the overall immune microenvironment score and the immune score in both datasets (**Figures 3b, c**). This suggests that patients with high RNF220 expression may exhibit immune evasion within the tumor microenvironment. Analysis using the CIBERSORT algorithm also revealed a stronger association between RNF220 and immune features in AML (**Figure 3d**). Specifically, AML patients with high RNF220 expression displayed decreased CD8^+^ T-cell infiltration and suppressed NK cell activity (**Figure 3d**), alongside an increased polarization of macrophages toward the M2 phenotype (**Figures 3e, f**). These findings further indicate an immunosuppressive tumor microenvironment in AML patients with high RNF220 expression (37). Subsequent cell-type classification analysis using MCP-counter corroborated the stronger immune correlation of RNF220 in AML and identified decreased CD8^+^ T cells and increased endothelial cells in patients with high RNF220 expression (**Figures 3g, h**). Furthermore, we analyzed the correlation between RNF220 and tumor immune evasion-related genes across pan-cancer datasets. This revealed that the association of RNF220 with immune evasion may be broadly conserved (**Supplementary Figure S3a**). Considering that tumor mutational burden (TMB) is also an integral component of the tumor immune landscape, we further assessed the correlation between TMB and RNF220 expression. The analysis demonstrated a moderate negative correlation between TMB and RNF220 expression in AML (**Supplementary Figures S3b**, **4C**).

**Figure 4:**
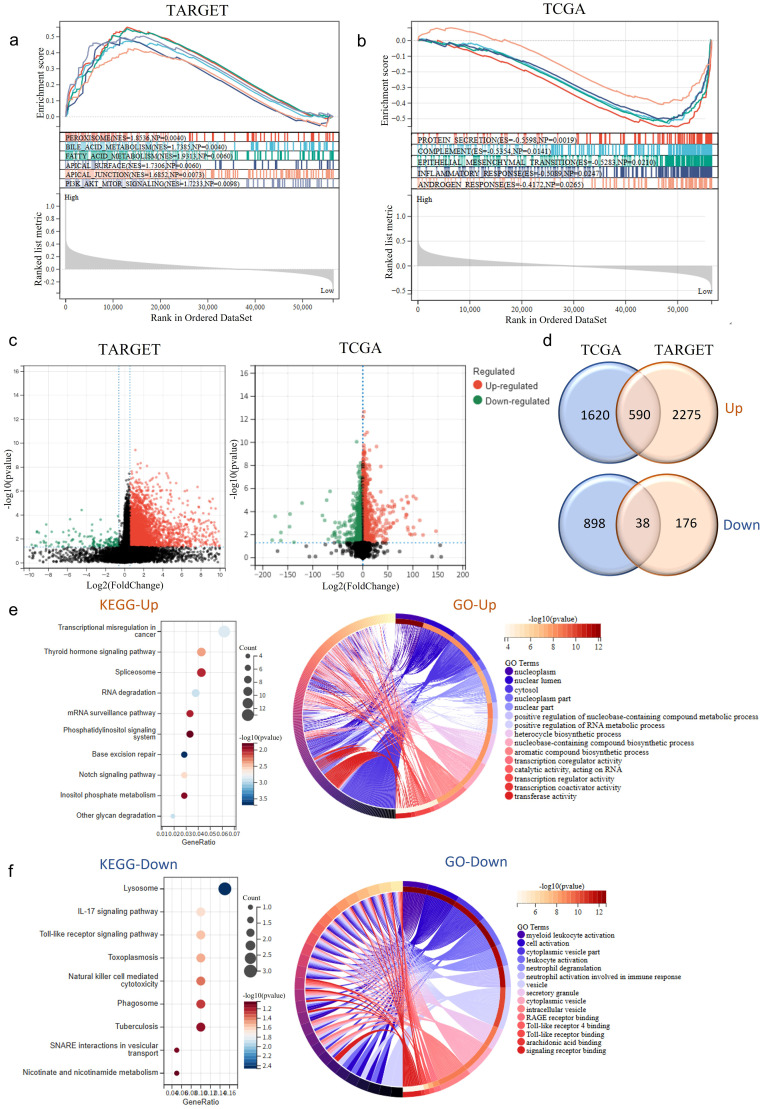
RNF220 expression alters tumor biological behavior. **(a)** Gene set enrichment analysis (GSEA) of HALLMARK pathways in TARGET-LAML patients stratified by RNF220 expression levels (high vs. low). **(b)** GSEA of HALLMARK pathways in TCGA-LAML patients stratified by RNF220 expression (high vs. low). **(c)** Volcano plots showing differentially expressed genes (DEGs) between RNF220 high- and low-expression groups in the two datasets. **(d)** Venn diagrams identifying overlapping upregulated/downregulated DEGs between the two datasets. **(e)** KEGG and GO functional enrichment analysis of upregulated genes in RNF220 high-expression groups. **(f)** KEGG and GO functional enrichment analysis of downregulated genes in RNF220 high-expression groups.

### RNF220 expression alters tumor biological behavior

To further investigate the impact of RNF220 on tumor cells, we stratified patients from the TCGA-LAML and TARGET-LAML cohorts into high- and low-expression groups based on RNF220 levels. GSEA of HALLMARKs pathways revealed that tumors with high RNF220 expression exhibited enhanced lipid and bile acid metabolism, as well as increased cell adhesion and EMT activity (**Figures 4a, b**). This suggests a higher malignant potential in patients with elevated RNF220 expression. Subsequently, we performed differential gene expression analysis between RNF220 high- and low-expression groups in each dataset (**Figure 4c**). KEGG and GO enrichment analyses of the upregulated and downregulated genes in each dataset are shown in **Supplementary Figures S4a–d**. Taking the intersection of upregulated and downregulated genes from both datasets yielded 590 positively correlated genes and 38 negatively correlated genes (**Figure 4d**). Enrichment analysis of the commonly upregulated genes identified functions primarily related to tumor-associated transcriptional regulation, RNA splicing, metabolism, and transport processes (**Figure 4e**). Conversely, the commonly downregulated genes were enriched in inflammatory and immune signaling pathways, as well as myeloid cell activation processes (**Figure 4f**). These findings suggest that high RNF220 expression is associated with aberrant tumor-related transcriptional regulation.

### RNF220 promotes AML cell proliferation

**Figure 5:**
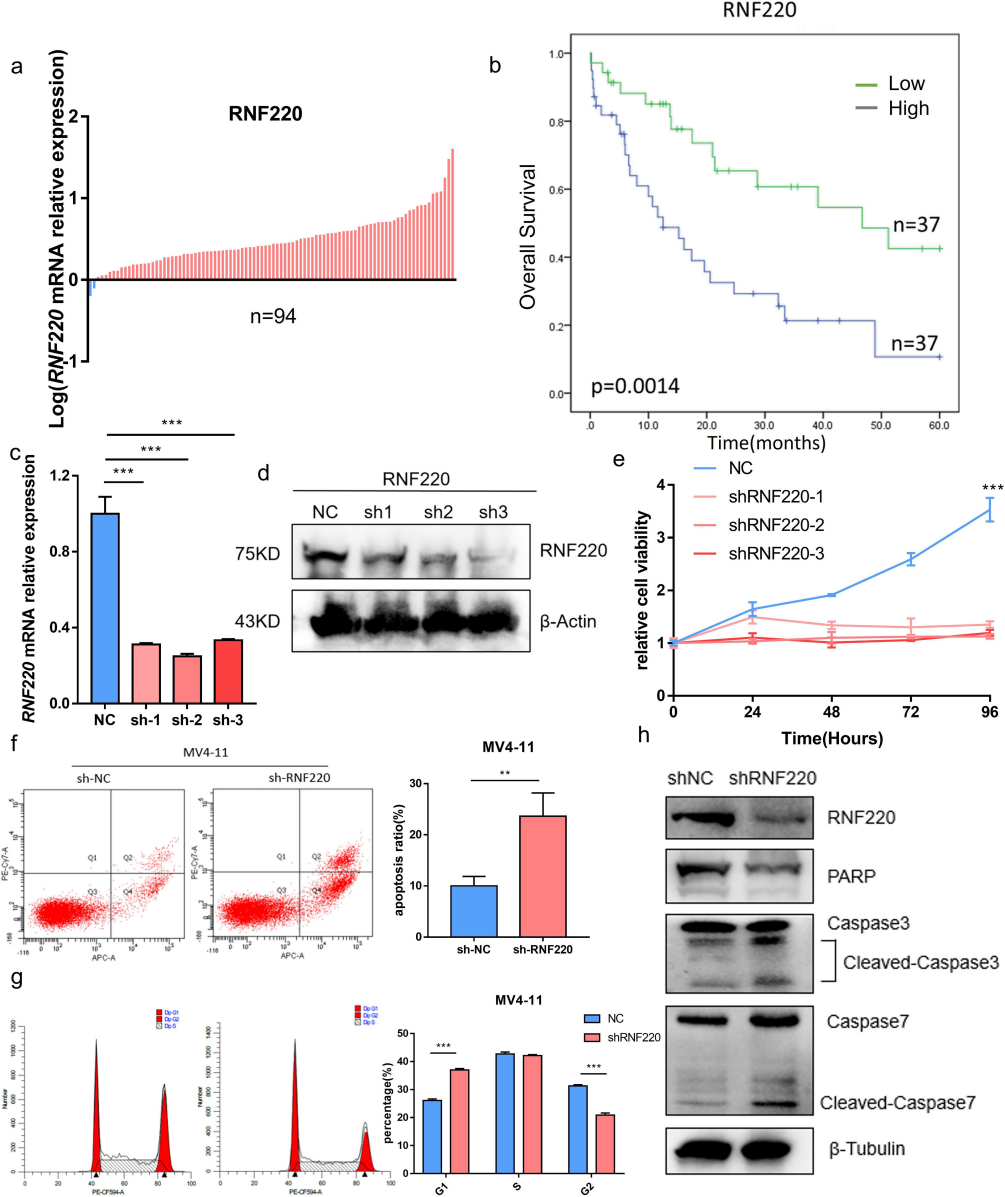
RNF220 promotes AML cell proliferation. **(a)** qPCR analysis showing RNF220 expression levels in AML patient tissues relative to MV4–11 cells. Blue: lower than MV4-11; red: higher than MV4-11. **(b)** Kaplan–Meier survival curve demonstrating prognostic impact of RNF220 in our cohort of 74 AML patients. **(c)** qPCR validation of RNF220 knockdown efficiency in THP-1 cells transduced with three distinct shRNAs. **(d)** Western blot analysis confirming RNF220 protein reduction in THP-1 cells following transduction with three shRNAs. **(e)** CCK-8 proliferation assays in RNF220-knockdown THP-1 cells measured at 0, 24, 48, 72, and 96 **(h)** The cellular proliferation rate was significantly reduced after knockdown of RNF220. **(f)** Flow cytometric analysis of apoptosis in MV4–11 cells after RNF220 knockdown. The apoptosis rate was increased after RNF220 knockdown. **(g)** Cell cycle distribution analysis by flow cytometry in RNF220-knockdown MV4–11 cells. Cells in the G1 phase were increased and decreased in the G2 phase. **(h)** Western blot detection of RNF220, PARP, and cleaved Caspase-3/7 in MV4–11 cells following RNF220 knockdown. Data are expressed as mean ± SEM (error bars). Statistical analyses used Student’s *t*-test and Kaplan−Meier survival analysis. *p < 0.05, **p < 0.01, and ***p < 0.001.

qPCR analysis of RNF220 expression in 94 AML patients, using the MV4–11 cell line (high RNF220 expression) as a baseline, revealed that most patients exhibited RNF220 levels higher than this baseline (**Figure 5a**). Patients’ information is shown in **Table 1**. Excluding 20 patients without treatment or receiving hematopoietic stem cell transplantation (HSCT), prognostic analysis confirmed that high RNF220 expression was associated with poor prognosis (**Figure 5b**). Then, we performed univariate and multivariate Cox regression analyses incorporating RNF220 expression levels and relevant clinical data to assess their prognostic significance. These analyses confirmed that RNF220 serves as an independent risk factor for prognosis in AML patients (**Table 2**). Next, we performed knockdown of RNF220 using shRNA in THP-1 cells (**Figures 5c, d**), resulting in reduced cell proliferation, as measured by the CCK-8 assay (**Figure 5e**). Furthermore, RNF220 knockdown in MV4–11 cells increased apoptosis (**Figure 5f**) and induced cell cycle arrest at the G1 phase (**Figure 5g**). Western blot analysis showed increased levels of cleaved Caspase-3 and Caspase-7 upon RNF220 knockdown, but no corresponding increase in cleaved PARP, suggesting activation of a non-canonical apoptotic pathway (**Figure 5h**).

**Table 1:** Association between RNF220 expression levels and pretreatment clinical characteristics of AML patients.

Characteristic	High-expression group (*n* = 47)	Low-expression group (*n* = 47)	*P*-value
Age (years), mean ± SD	45.28 ± 16.81	43.47 ± 16.69	>0.05
Gender, *n*			
Male	26	25	>0.05
Female	21	22	
WBC (×10^9^/L), mean ± SD	66.42 ± 85.62	40.43 ± 46.02	>0.05
Hb (g/L), mean ± SD	83.80 ± 24.45	84.59 ± 21.39	>0.05
PLT (×10^9^/L), mean ± SD	51.74 ± 52.81	78.45 ± 99.46	>0.05
BM blasts (%), mean ± SD	71.60 ± 1.46	62.53 ± 20.32	**<0.05**
Cytogenetic risk, *n*			
Unknown	3	3	>0.05
Favorable	3	1	
Intermediate	37	40	
Adverse	4	3	
CR rate, %	76.2%	81.4%	>0.05

The bold values means "it is significant" (p<0.05).

**Table 2:** Univariate and multivariate Cox regression analysis of prognostic factors in AML patients.

Variable	Univariate analysis			Multivariate analysis		
	*P*-value	HR	95% CI	*P*-value	HR	95% CI
Age	0.037	0.518	0.279–0.961	0.290	0.705	0.370–1.346
Gender	0.005	0.382	0.195–0.751	0.052	0.492	0.241–1.006
WBC (×10^9^/L)	**0.001**	1.008	1.003–1.012	**0.019**	1.006	1.001–1.010
Hb (g/L)	0.411	0.994	0.981–1.008	NS	–	–
PLT (×10^9^/L)	0.523	0.998	0.992–1.004	NS	–	–
BM blasts (%)	0.153	1.013	0.995–1.031	NS	–	–
RNF220 expression	**0.002**	2.809	1.451–5.438	**0.023**	2.236	1.115–4.483

NS, not selected.The bold values means "it is significant" (p<0.05).

### RNF220 is associated with glycolysis and phenylalanine metabolism

**Figure 6:**
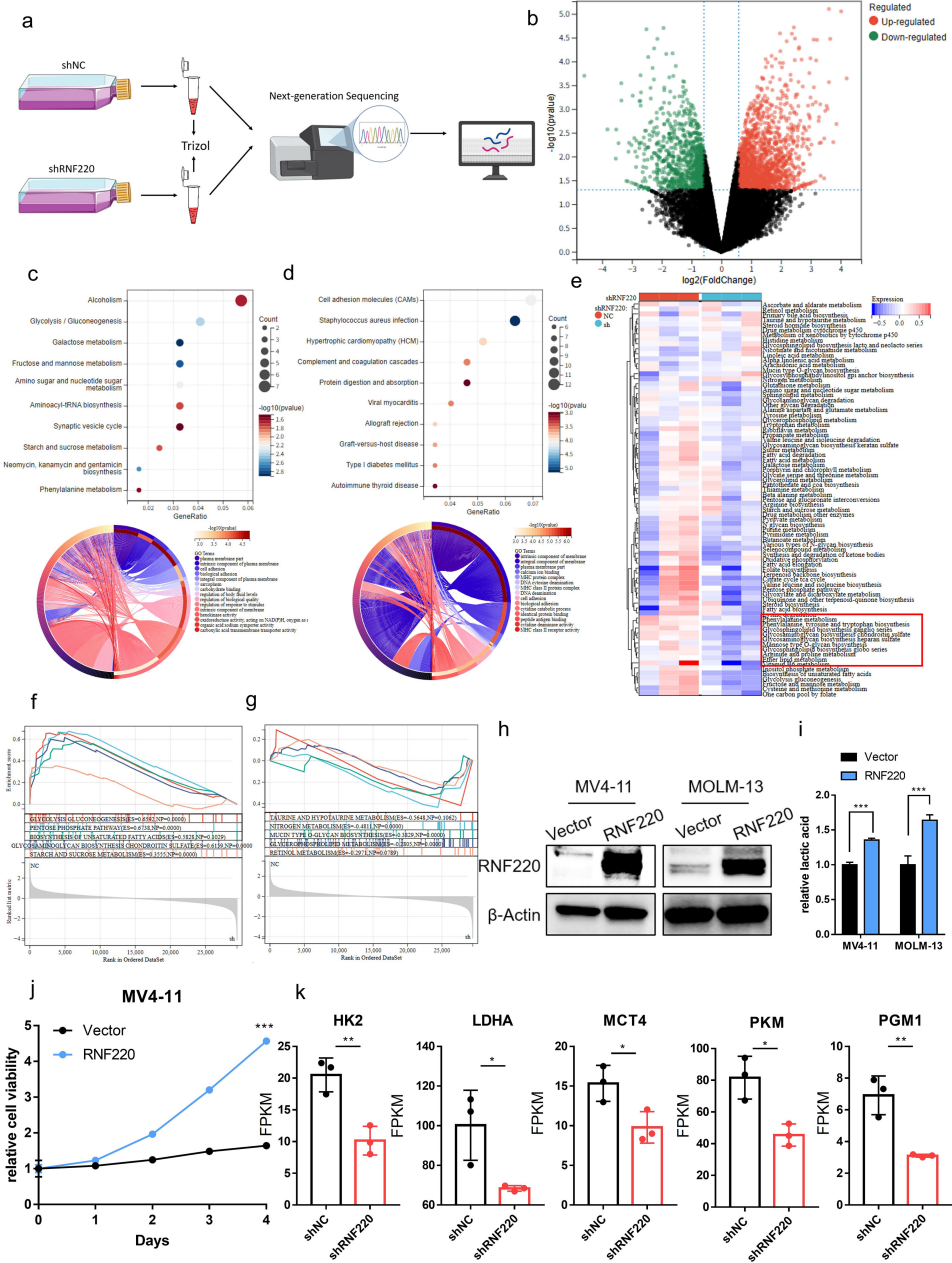
RNF220 is associated with glycolysis and phenylalanine metabolism. **(a)** Workflow diagram of transcriptome sequencing following RNF220 knockdown in MV4–11 cells. **(b)** Volcano plot showing differentially expressed genes after RNF220 knockdown in MV4–11 cells. **(c)** KEGG and GO enrichment analyses of downregulated genes in RNF220-knockdown MV4–11 cells. **(d)** KEGG and GO enrichment analyses of upregulated genes in RNF220-knockdown MV4–11 cells. **(e)** Heatmap of GSVA scores for metabolic pathways in control vs. RNF220-knockdown MV4–11 cells. **(f)** GSEA demonstrating downregulated metabolic pathways in RNF220-knockdown MV4–11 cells. **(g)** GSEA revealing upregulated metabolic pathways in RNF220-knockdown MV4–11 cells. **(h)** Western blot analysis confirming RNF220 protein overexpression in MV4–11 and MOLM-13 cells. **(i)** The lactic acid level in culture medium was increased after overexpressing RNF220 in MV4–11 and MOLM-13 cells. **(j)** CCK-8 proliferation assays in RNF220 overexpression in MV4–11 cells measured at 0, 24, 48, 72, and 96 **(h)** The cellular proliferation rate was significantly increased after overexpression of RNF220. **(k)** FPKM of HK2, LDHA, MCT4, PKM, and PGM1 in the RNA-seq of knocking down RNF220 in MV4–11 cells. *p < 0.05, **p < 0.01, ***p < 0.001, and ****p < 0.0001.

To further investigate RNF220’s function in AML cells, we performed RNA sequencing on MV4–11 cells following RNF220 knockdown (**Figures 6a, b**). Gene Ontology (GO) enrichment analysis of downregulated genes identified significant decreases in glycolysis and phenylalanine metabolism pathways (**Figure 6c**), while upregulated genes were primarily enriched in macromolecule metabolic processes such as protein and DNA metabolism (**Figure 6d**). To validate the link between RNF220 and metabolism, we collected metabolic pathways from the BioCyc database and calculated gene set variation analysis (GSVA) scores. All the metabolic pathways are shown in **Supplementary Table S2**. This confirmed a significant decrease in glycolysis and phenylalanine metabolism scores after RNF220 knockdown (**Figure 6e**). GSEA further supported these findings (**Figures 6f, g**). Additionally, correlation analysis (**Supplementary Figures S5a, b**) and GSEA (**Supplementary Figures S5c, d**) of TCGA-LAML and TARGET-LAML patient data demonstrated positive correlations between RNF220 expression and both glycolysis and phenylalanine metabolism pathways. Subsequently, we overexpressed RNF220 in MV4–11 and MOLM13 cells, with the overexpression efficiency shown in **Figure 6h**. Following this, lactate levels in the cell culture medium were measured and found to be elevated upon RNF220 overexpression (**Figure 6i**), along with an increase in cell proliferation capacity (**Figure 6j**). Furthermore, we analyzed the transcriptional levels of key glycolysis-related molecules in sequencing data after RNF220 knockdown and observed decreased expression of HK2, LDHA, MCT4, PKM, and PGM1 following RNF220 knockdown (**Figure 6k**), while no significant differences were detected in the expression of PFKP and MCT1 (**Supplementary Figure S5e**).

### FOXA1 is an upstream negative transcriptional regulator of RNF220

**Figure 7:**
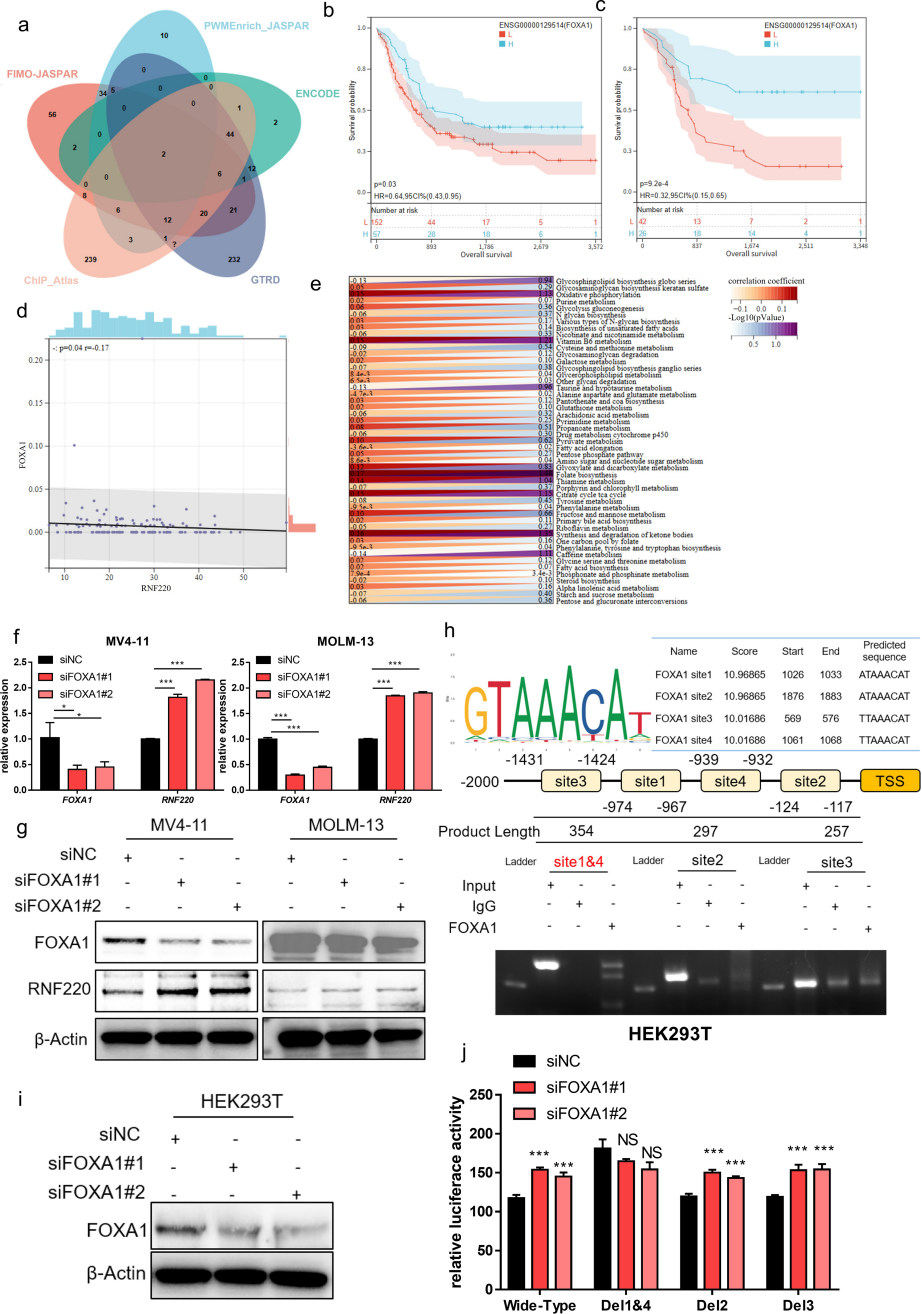
FOXA1 is an upstream negative transcriptional regulator of RNF220. **(a)** Venn diagram showing overlapping transcription factors predicted by five independent datasets to regulate RNF220 upstream. **(b)** Kaplan–Meier curve demonstrating the impact of FOXA1 expression on overall survival (OS) in the TCGA-LAML cohort. **(c)** Kaplan–Meier curve showing the effect of FOXA1 on OS in TCGA-LAML after excluding samples with zero expression. **(d)** Scatter plot illustrating the correlation between FOXA1 and RNF220 expression in TCGA-LAML. **(e)** Heatmap depicting the correlations between FOXA1 expression and metabolic signaling pathways in TCGA-LAML. Statistical analyses used non-paired Wilcoxon rank sum and signed rank tests and Kaplan−Meier survival analysis. **(f)** The mRNA level of FOXA1 and RNF220 after knocking down FOXA1 using siRNA in MV4–11 and MOLM-13 cells detected by qPCR. The mRNA of RNF220 was increased after knocking down FOXA1. **(g)** The protein level of FOXA1 and RNF220 after knocking down FOXA1 using siRNA in MV4–11 and MOLM-13 cells detected by Western blot. The protein level of RNF220 was increased after knocking down FOXA1. **(h)** The ChIP-PCR assay of FOXA1 in MV4–11 cell. **(i)** Western blot analysis confirming FOXA1 protein reduction in HEK293T cells following transduction with two siRNAs. **(j)** Luciferase reporter assays were performed in FOXA1-knockdown HEK293T cells transfected with wild-type and various mutant RNF220 promoter reporter plasmids. *p < 0.05, **p < 0.01, ***p < 0.001, and ****p < 0.0001.

**Figure 8:**
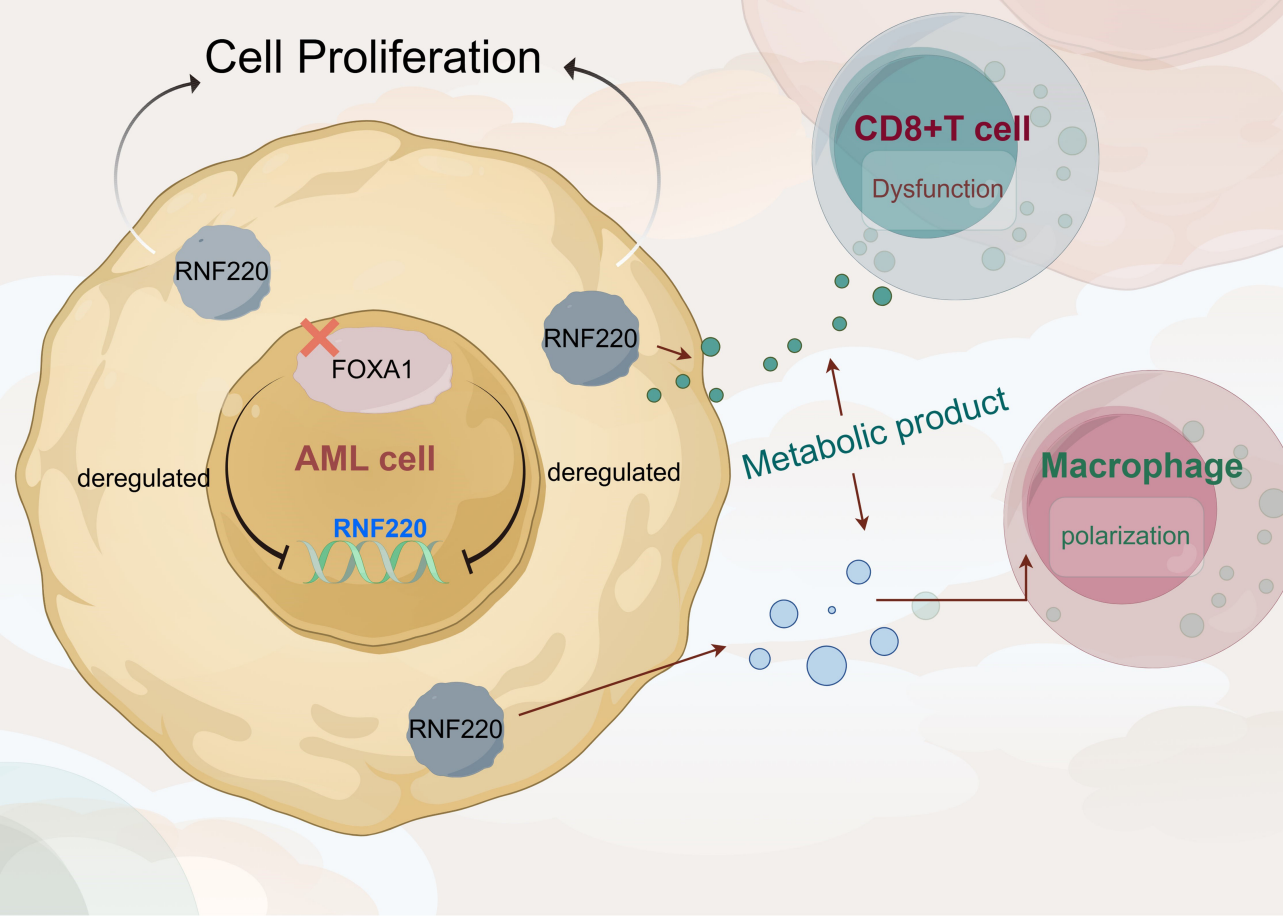
RNF220 coordinates leukemic metabolism and immunosuppression to accelerate AML progression.

To elucidate the cause of elevated RNF220 expression in AML, we predicted potential transcriptional regulators of RNF220 using five transcription factor (TF) databases (FIMO_JASPAR, PWMEnrich_JASPAR, ENCODE, GTRD, ChIP_Atlas). Intersection of the results identified two candidate TFs: FOXA1 and FOXA2 (**Figure 7a**). Expression analysis in the TCGA-LAML dataset showed that *FOXA1* expression was lower in AML samples compared to normal tissue, while *FOXB1* was higher. In contrast, *FOXA1* expression was higher in ALL samples, and *FOXA2* showed no significant difference (**Supplementary Figures S6a, b**). Due to a substantial number of patients showing zero expression for *FOXA1* and *FOXA2*, survival analysis was performed both conventionally and after excluding patients with zero expression. Regardless of the analytical approach, high *FOXA1* expression consistently predicted a favorable prognosis (**Figures 7b, c**), whereas *FOXA2* expression showed no significant association with survival (**Supplementary Figures S6c, d**). Correlation analysis revealed a significant negative correlation between *FOXA1* and *RNF220* expression (**Figure 7d**) but no significant correlation between *FOXA2* and *RNF220* (**Supplementary Figure S6e**). These results suggest that FOXA1 acts as a negative transcriptional regulator of *RNF220*. Further metabolic correlation analysis showed that the association of *FOXA1* with metabolic pathways was inversely related to that of *RNF220* (**Figure 7e**), providing additional evidence supporting FOXA1 as a negative transcriptional regulator of RNF220. Subsequently, we knocked down FOXA1 in MV4–11 and MOLM-13 cell lines and observed an increase in RNF220 expression at both the transcriptional (**Figure 7f**) and protein levels (**Figure 7g**). We then predicted potential FOXA1 binding sites within the RNF220 promoter region using the JASPAR database and performed a ChIP assay in MV4–11 cells. Due to the close proximity of predicted site 1 and site 4, it was not feasible to design independent primers to distinguish them; thus, they were amplified together in a single PCR product. The ChIP assay demonstrated that FOXA1 binds to site 1 (**Figure 7h**). Further luciferase reporter assays conducted in FOXA1-knockdown HEK293T cells (**Figure 7i**) confirmed that the genomic region containing both site 1 and site 4 is indeed bound by FOXA1 (**Figure 7j**). A schematic diagram of the proposed mechanistic model in this study is presented in **Figure 8**.

## Discussion

This study builds upon our group’s previous findings to explore RNF220 expression across pan-cancers. Survival analysis established its prognostic significance in AML, while analysis of public single-cell RNA-seq datasets revealed a positive correlation between RNF220 and malignant biological behaviors in AML, suggesting RNF220 may directly regulate tumor proliferation and dedifferentiation within neoplastic cells. Subsequently, we investigated RNF220’s relationship with the tumor immune microenvironment, where RNF220-high patients exhibited features of immune exhaustion and evasion characterized by decreased overall immune score, reduced CD8^+^ T-cell infiltration, impaired NK cell activity with increased dormant NK populations, and M0-to-M2 macrophage polarization with suppressed phagocytic capacity. These alterations indicate that RNF220 overexpression induces immune escape—a contributor to poor prognosis.

To elucidate mechanisms, we stratified public AML datasets by RNF220 expression and performed functional enrichment analyses, revealing that RNF220 promotes oncogenesis through aberrant transcriptional regulation that disrupts RNA/protein synthesis and transport pathways. Validation in our local AML cohort confirmed elevated RNF220 mRNA as an independent poor prognostic factor. *In vitro* RNF220 knockdown suppressed proliferation, increased apoptosis, and induced cell cycle arrest, with subsequent RNA-seq identifying upregulated glycolysis and phenylalanine metabolism—confirmed in public datasets.

Glycolysis provides critical energy in hypoxic tumors and promotes oncogenesis (38, 39), while also inducing immune dysfunction and evasion in the microenvironment (40–42), potentially explaining RNF220-mediated immunosuppression. Although phenylalanine metabolism’s role remains unclear, it may represent a metabolic by-product or immune-metabolic pathway requiring future investigation. Regarding RNF220 upregulation, multi-database analysis identified FOXA1 as a negative transcriptional regulator—this epigenetic modulator directly binds androgen receptor promoters (43), serves as an ER^+^ breast cancer biomarker (44), and drives progression in prostate/breast cancers via mutational activation (45–47). Its newly identified role in suppressing AML through RNF220 inhibition merits mechanistic exploration. Study limitations include unresolved metabolic regulation mechanisms, lack of proteomics for E3 substrate identification, and absence of *in vivo* validation. These will be taken into consideration in further investigations.

Certainly, this study has several limitations. First, the *in vitro* experiments were confined to AML cells themselves. Although we confirmed the impact of RNF220 on cell proliferation, we did not perform co-culture assays to investigate its effect on microenvironmental cells in AML, nor did we conduct *in vivo* experiments to examine the influence of RNF220 on the immune microenvironment. The conclusions regarding the immune-related effects of RNF220 were primarily based on correlative transcriptomic analysis and estimations from deconvolution algorithms, which do not fully capture the functional state of the immune microenvironment. More functional studies are needed in the future to address this. Furthermore, although we detected changes in lactate levels in the cell culture medium after RNF220 overexpression, the conclusion regarding metabolic reprogramming largely relied on enrichment analysis. The underlying mechanisms also require further experimental exploration. Additionally, the local clinical cohort in this study was derived from a single center, with a relatively small sample size and potential selection bias. Multivariate analyses also did not adjust for confounding factors such as molecular subtypes and treatment strategies, which represents another limitation.

## Conclusion

Collectively, our integrated evidence establishes RNF220 as a FOXA1-suppressed independent prognostic biomarker that accelerates AML progression through glycolytic/phenylalanine metabolic reprogramming and immune-metabolic evasion.

## Data Availability

The datasets presented in this study can be found in online repositories. The names of the repository/repositories and accession number(s) can be found in the article/**Supplementary Material**.

## Supplementary material

Supplementary Figure 1
**(a-h)** Kaplan-Meier curves showing prognostic impact of RNF220 across various cancers. Cancer types are labeled above each panel.

Supplementary Figure 2
**(a-f)** Distribution of RNF220 expression across cell types in single-cell datasets. Left panels: malignant/non-malignant cell annotation; center panels: cell subpopulation clusters; right panels: RNF220 expression levels. Dataset names are indicated above panels.

Supplementary Figure 3
**(a)** Heatmap of correlations between RNF220 and immune-related genes in pan-cancer analysis. **(b)** Lollipop plot showing correlation between RNF220 and tumor mutational burden (TMB) across cancers. **(c)** Scatter plot demonstrating RNF220-TMB correlation in TCGA-LAML.

Supplementary Figure 4
**(a)** KEGG and GO enrichment of upregulated genes in RNF220-high TARGET-LAML cohort. **(b)** Enrichment of downregulated genes in RNF220-high TARGET-LAML. **(c)** Enrichment of upregulated genes in RNF220-high TCGA-LAML. **(d)** Enrichment of downregulated genes in RNF220-high TCGA-LAML.

Supplementary Figure 5
**(a)** Heatmap of RNF220-metabolic pathway correlations in TARGET-LAML. **(b)** Metabolic correlation heatmap in TCGA-LAML. **(c)** GSEA of metabolic pathways associated with RNF220 in TARGET-LAML. **(d)** Metabolic pathway GSEA in TCGA-LAML. **(e)** PKM and PGM1 in the RNA-seq of knocking down RNF220 in MV4–11 cell. NS, not significant.

Supplementary Figure 6
**(a)** Differential expression of FOXA1 genes between tumor and normal tissues in TCGA-LAML and TCGA-ALL datasets. **(b)** Differential expression of FOXA2 genes between tumor and normal tissues in TCGA-LAML and TCGA-ALL datasets **(c)** Kaplan-Meier curve showing impact of FOXA2 expression on OS in TCGA-LAML. **(d)** OS analysis after excluding FOXA2 non-expressing samples. **(e)** Scatter plot of FOXA2-RNF220 expression correlation in TCGA-LAML.
