# Supplementary figures and images for: Dual oncogenic role of RNF220 in AML: linking metabolic rewiring to cell proliferation and immune evasion
Source: Front Oncol. 2025 Oct 30;15:1670895. doi: 10.3389/fonc.2025.1670895 (PMC12611666; doi:10.3389/fonc.2025.1670895)

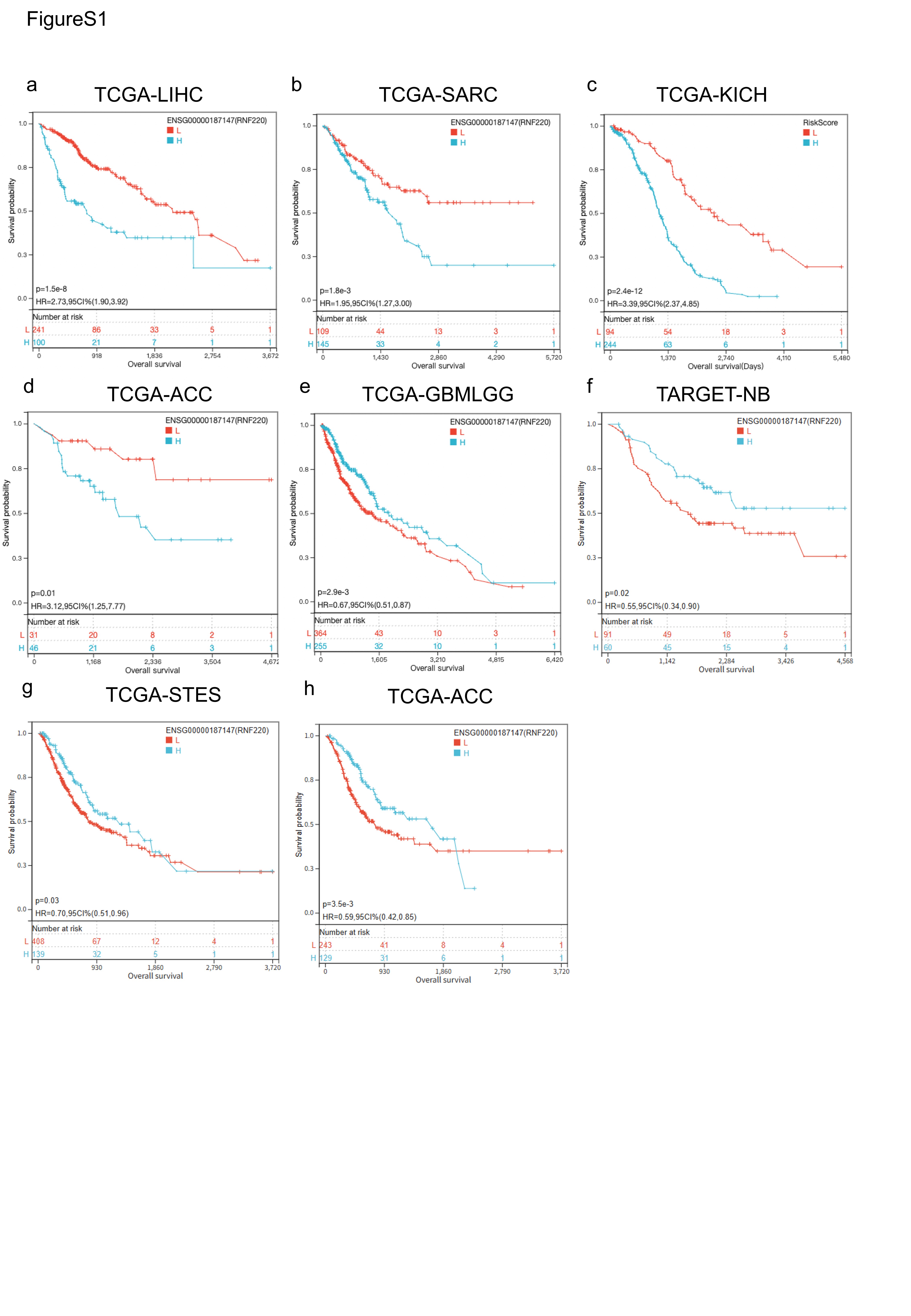

Supplement: Supplementary Figure 1 — (a-h) Kaplan-Meier curves showing prognostic impact of RNF220 across various cancers. Cancer types are labeled above each panel. [file Image1.jpeg]

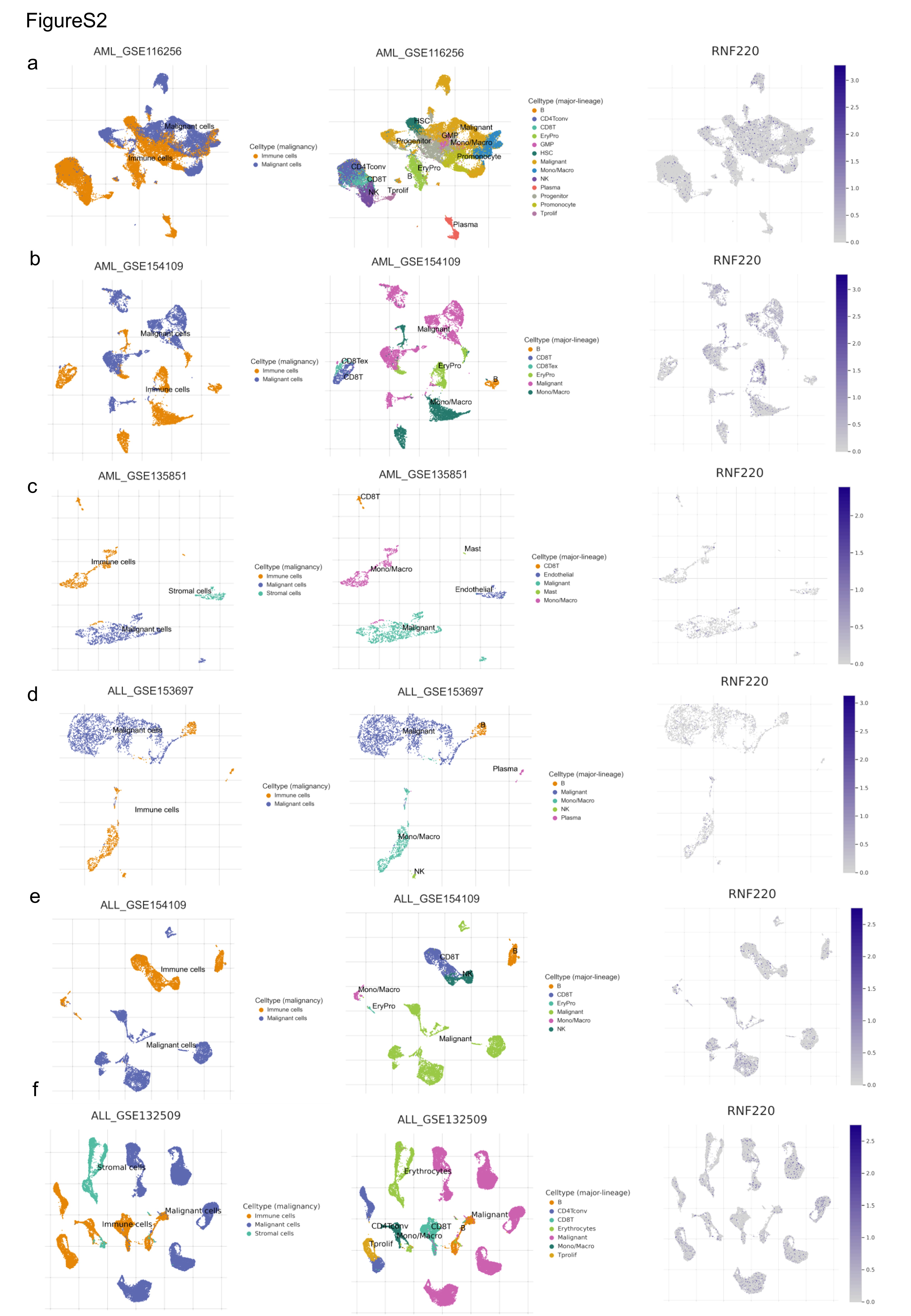

Supplement: Supplementary Figure 2 — (a-f) Distribution of RNF220 expression across cell types in single-cell datasets. Left panels: malignant/non-malignant cell annotation; center panels: cell subpopulation clusters; right panels: RNF220 expression levels. Dataset names are indicated above panels. [file Image2.jpeg]

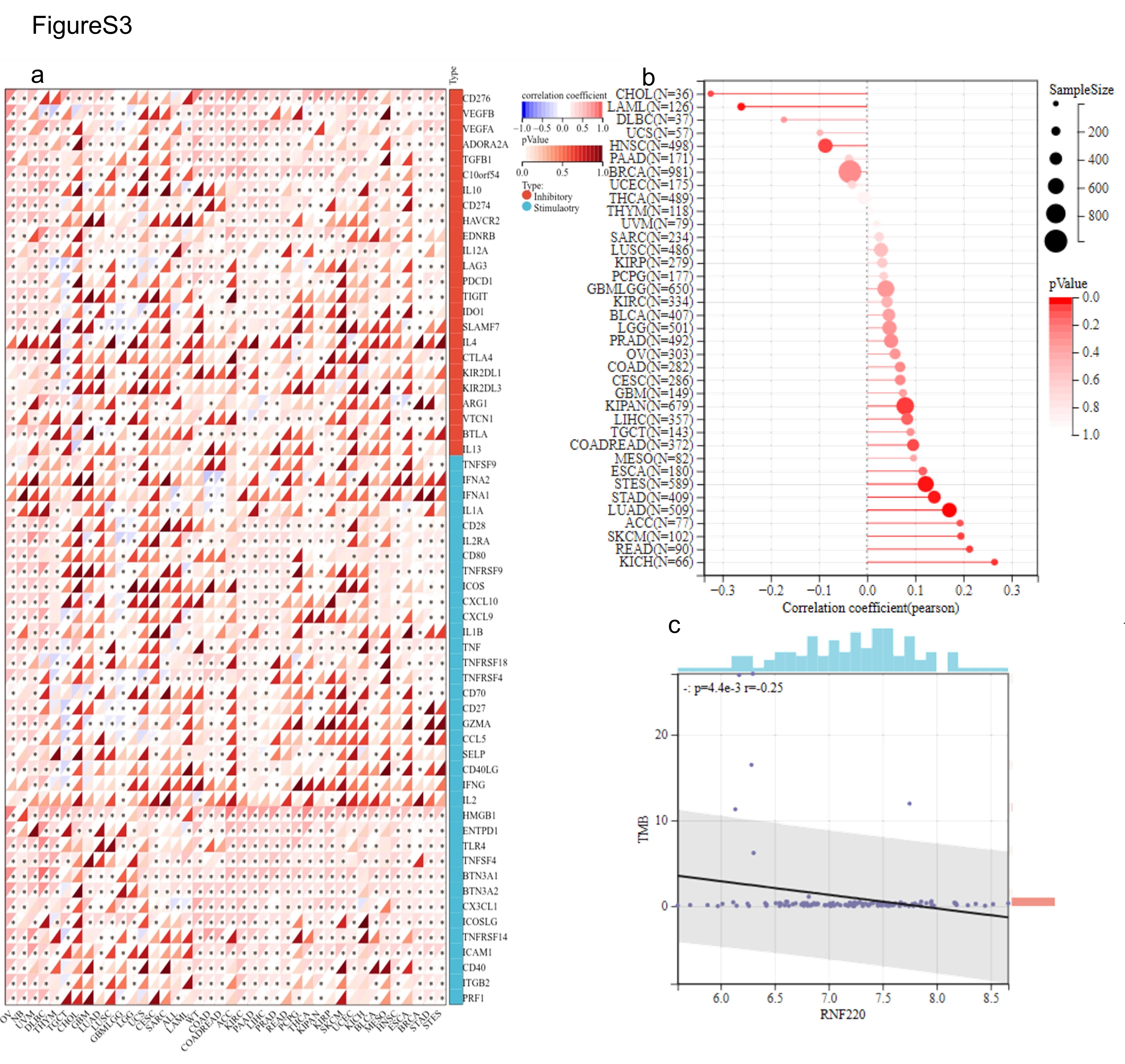

Supplement: Supplementary Figure 3 — (a) Heatmap of correlations between RNF220 and immune-related genes in pan-cancer analysis. (b) Lollipop plot showing correlation between RNF220 and tumor mutational burden (TMB) across cancers. (c) Scatter plot demonstrating RNF220-TMB correlation in TCGA-LAML. [file Image3.jpeg]

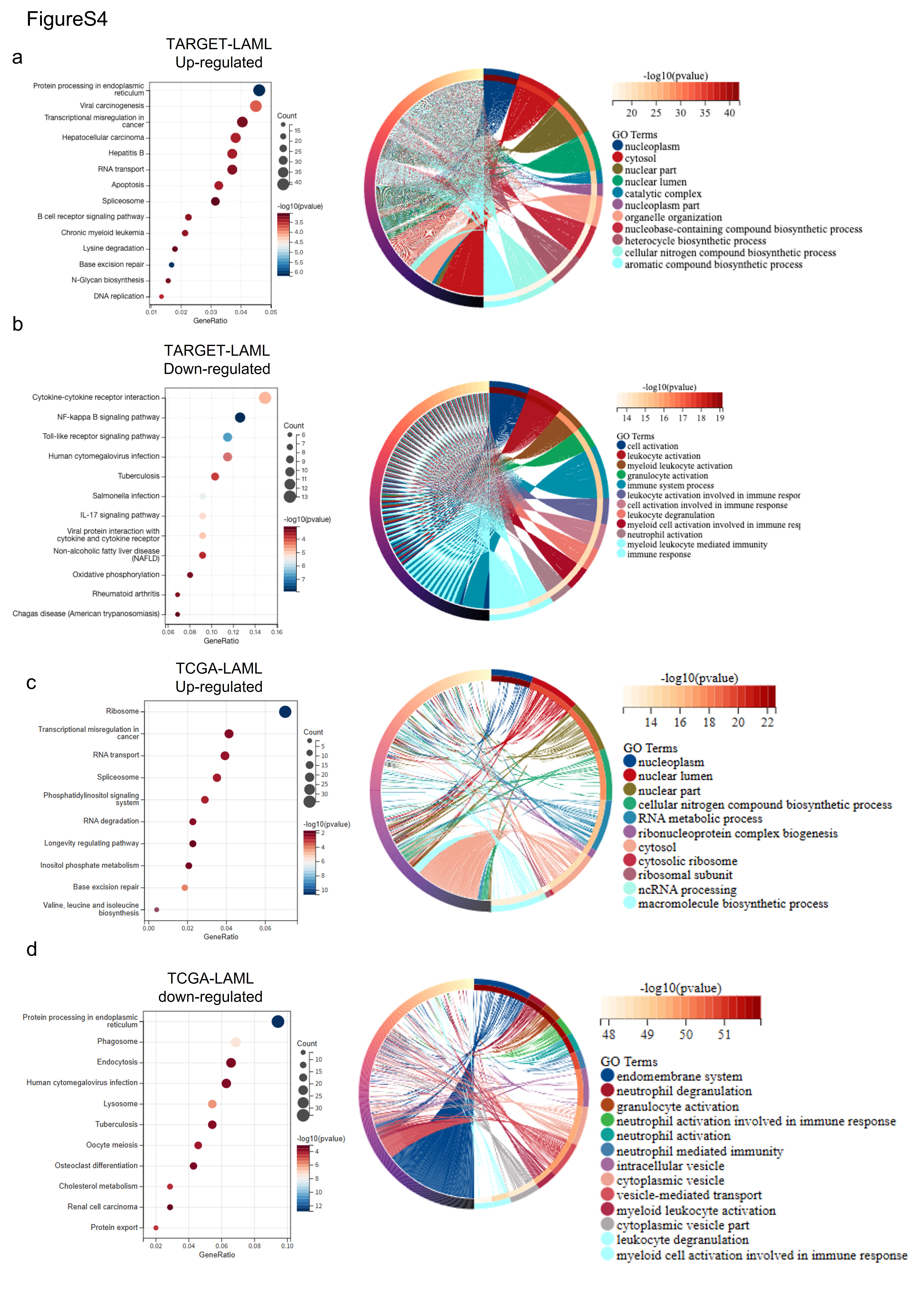

Supplement: Supplementary Figure 4 — (a) KEGG and GO enrichment of upregulated genes in RNF220-high TARGET-LAML cohort. (b) Enrichment of downregulated genes in RNF220-high TARGET-LAML. (c) Enrichment of upregulated genes in RNF220-high TCGA-LAML. (d) Enrichment of downregulated genes in RNF220-high TCGA-LAML. [file Image4.jpeg]

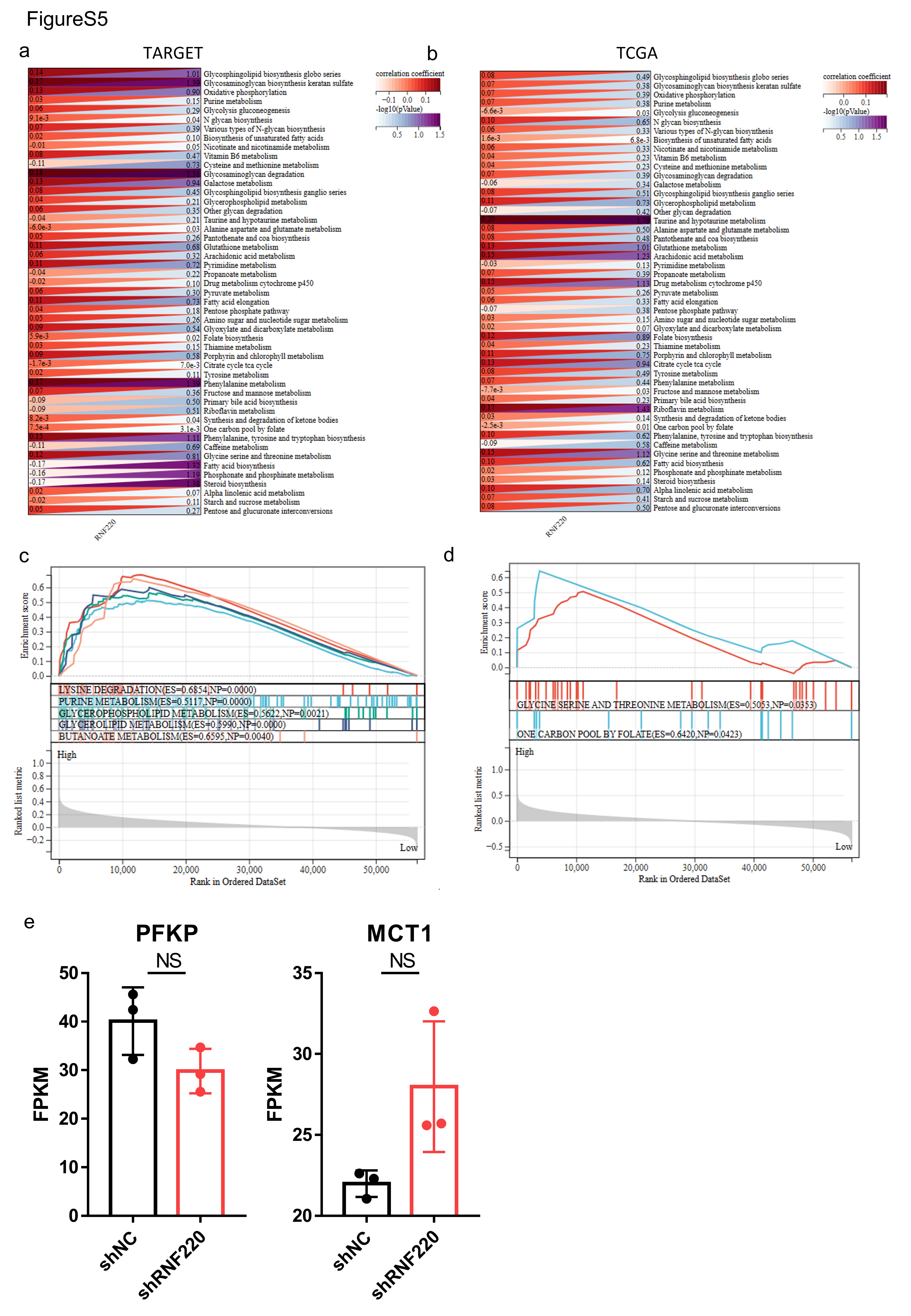

Supplement: Supplementary Figure 5 — (a) Heatmap of RNF220-metabolic pathway correlations in TARGET-LAML. (b) Metabolic correlation heatmap in TCGA-LAML. (c) GSEA of metabolic pathways associated with RNF220 in TARGET-LAML. (d) Metabolic pathway GSEA in TCGA-LAML. (e) PKM and PGM1 in the RNA-seq of knocking down RNF220 in MV4–11 cell. NS, not significant. [file Image5.jpeg]

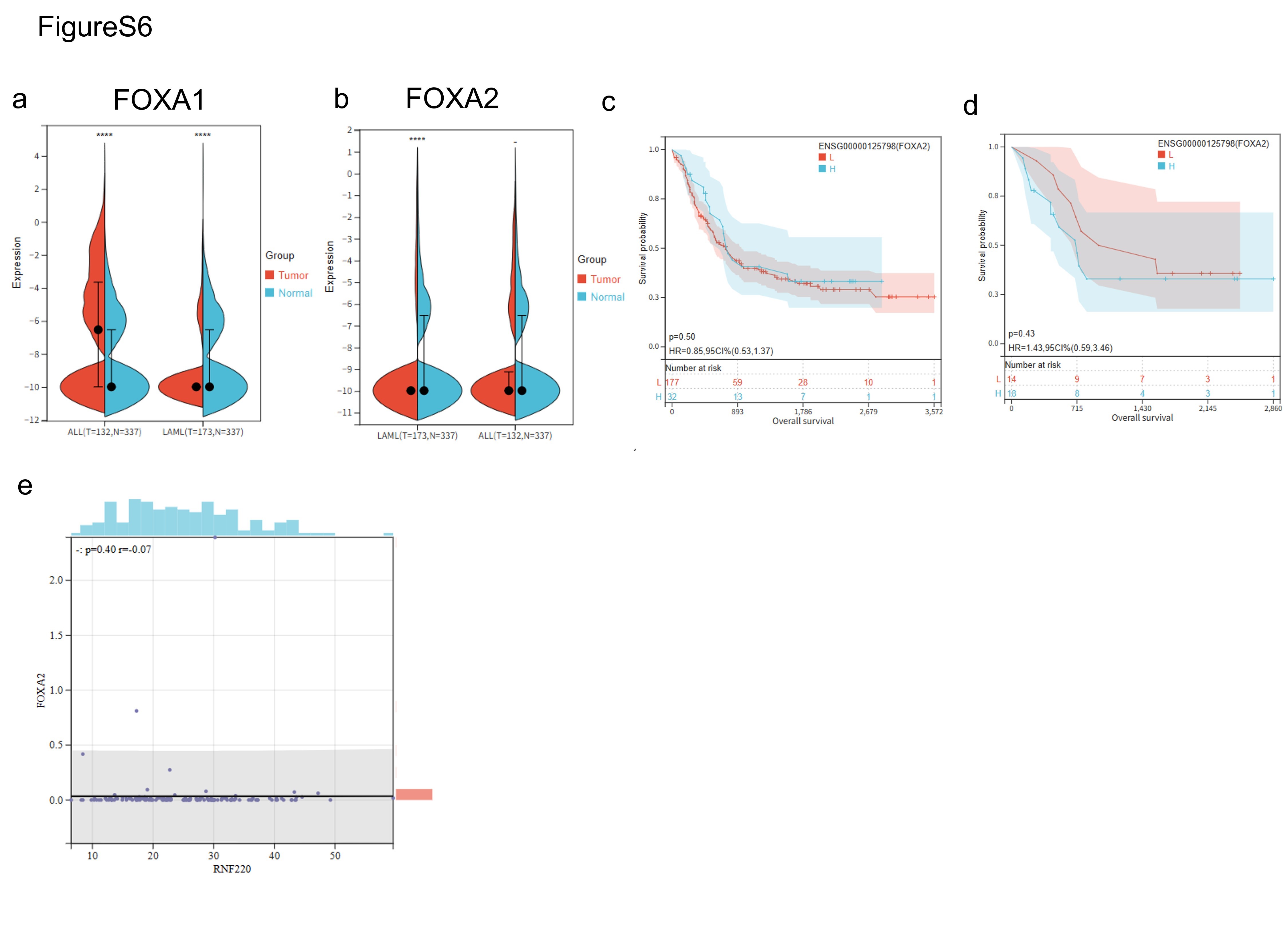

Supplement: Supplementary Figure 6 — (a) Differential expression of FOXA1 genes between tumor and normal tissues in TCGA-LAML and TCGA-ALL datasets. (b) Differential expression of FOXA2 genes between tumor and normal tissues in TCGA-LAML and TCGA-ALL datasets (c) Kaplan-Meier curve showing impact of FOXA2 expression on OS in TCGA-LAML. (d) OS analysis after excluding FOXA2 non-expressing samples. (e) Scatter plot of FOXA2-RNF220 expression correlation in TCGA-LAML. [file Image6.jpeg]
