# Supplementary material for: Dual oncogenic role of RNF220 in AML: linking metabolic rewiring to cell proliferation and immune evasion
Source: Front Oncol. 2025 Oct 30;15:1670895. doi: 10.3389/fonc.2025.1670895 (PMC12611666; doi:10.3389/fonc.2025.1670895)

Fig5d

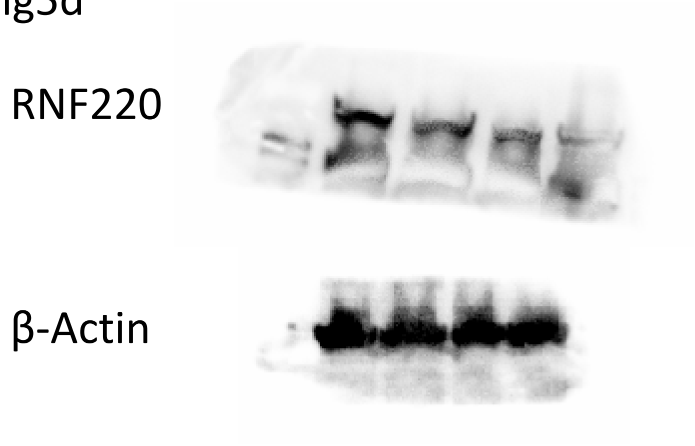

Fig5h

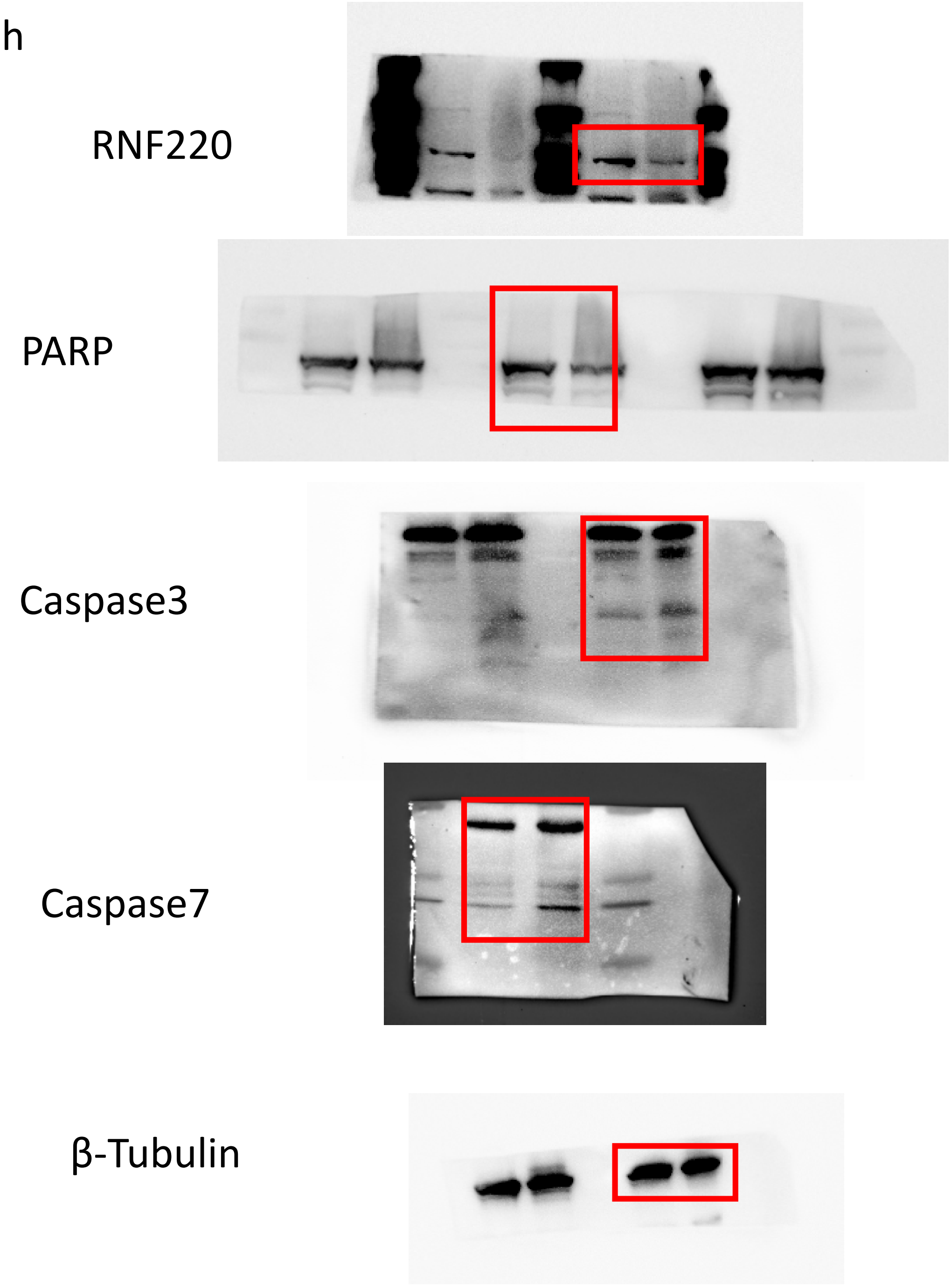

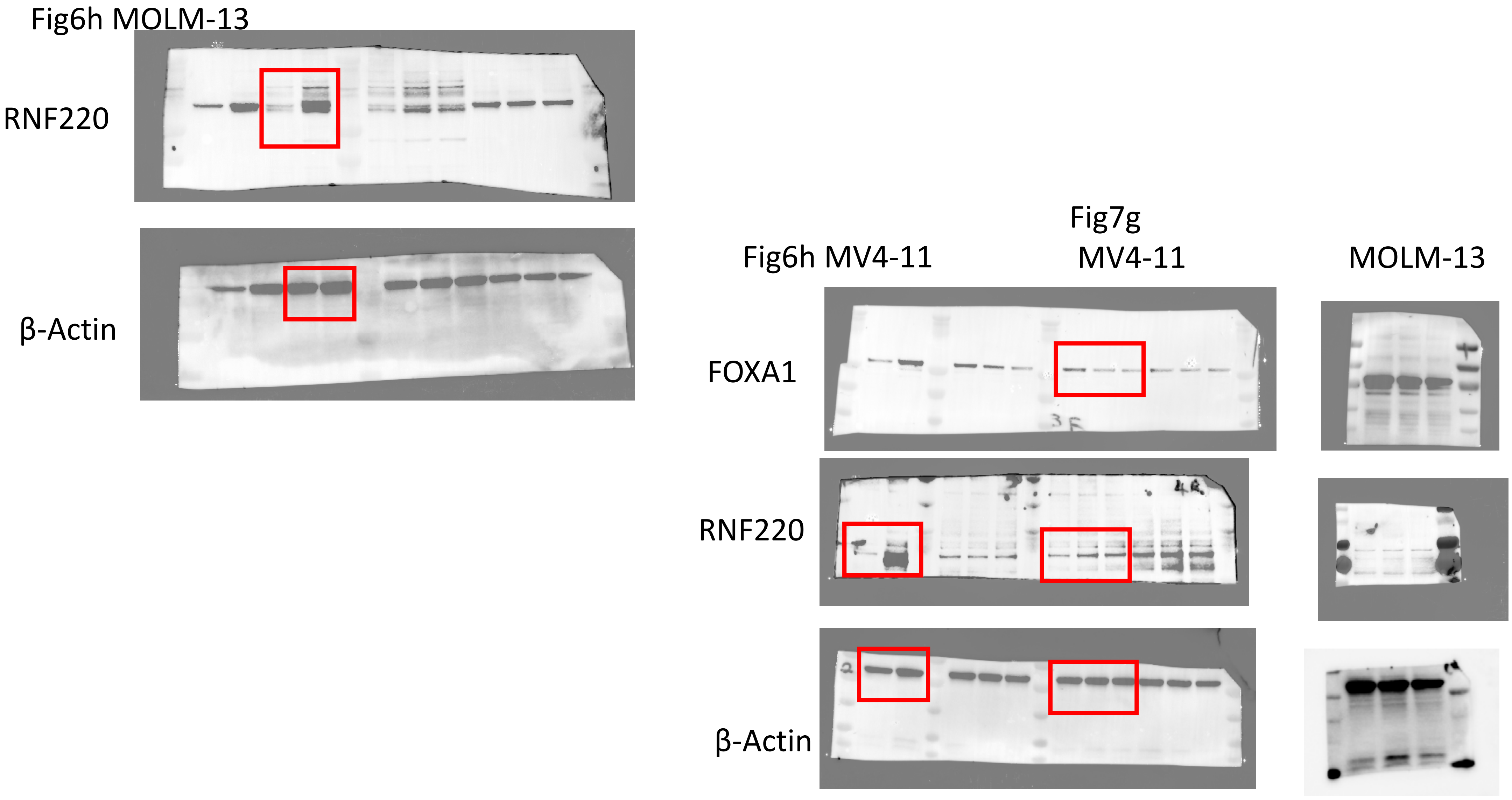
Fig6h MOLM-13
RNF220
β-Actin
Fig7g
Fig6h MV4-11
MV4-11
MOLM-13
FOXA1
RNF220
β-Actin

Fig7i HEK293T

FOXA1

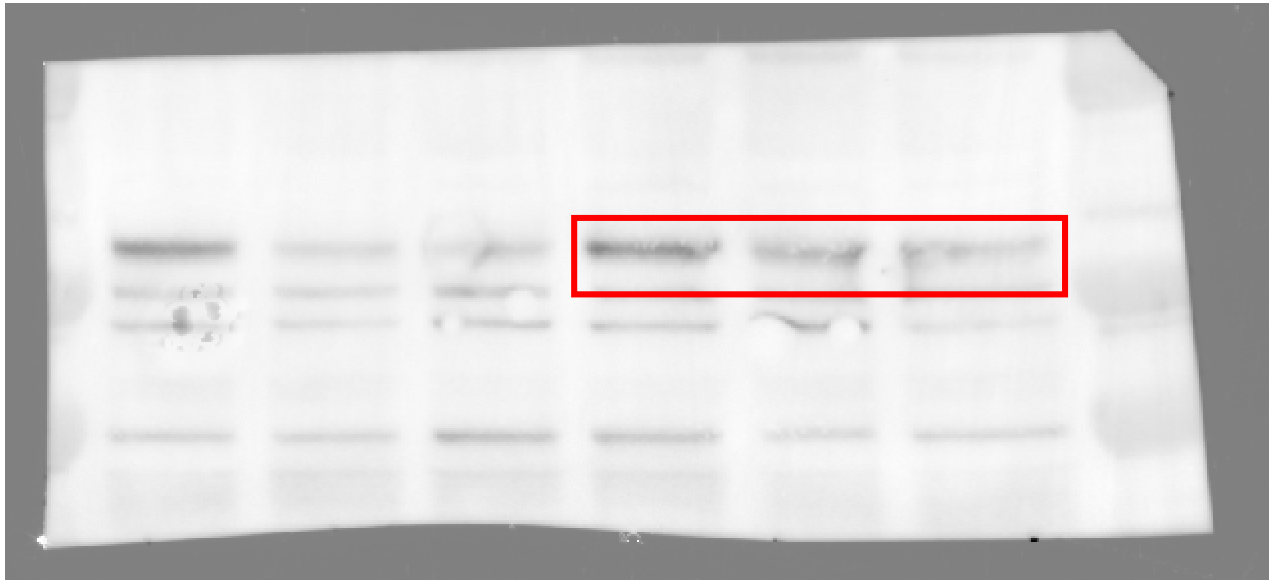

β-Actin

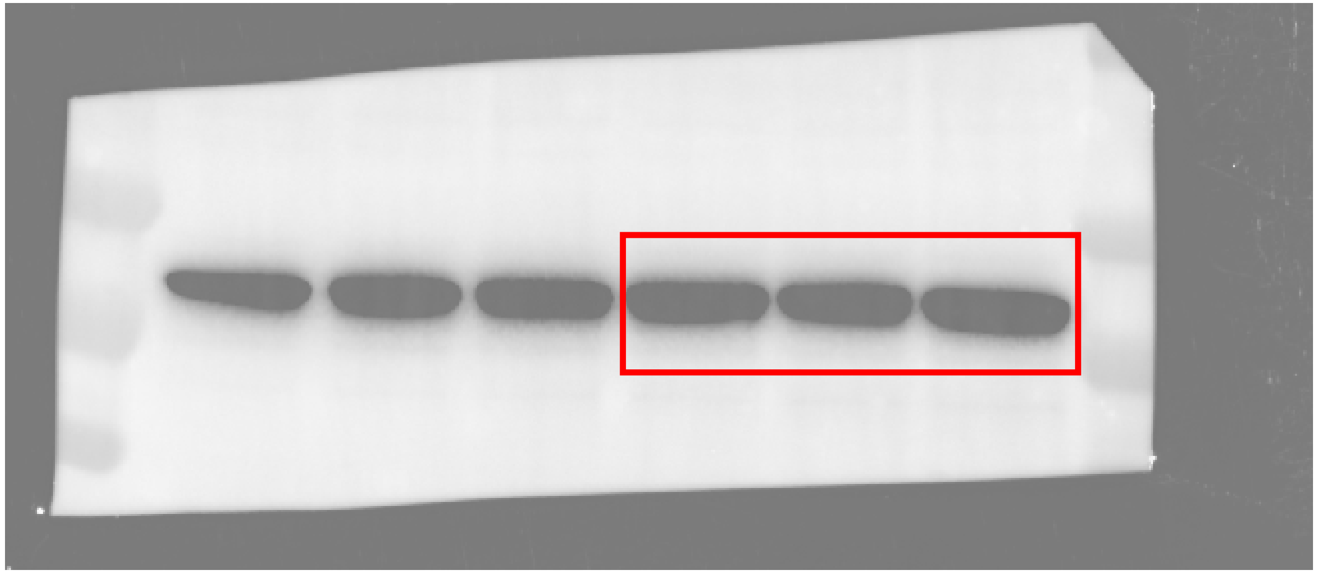

Supplement: Supplementary file 10 [file DataSheet1.pdf]
